# Ceramide as a Mediator of Non-Alcoholic Fatty Liver Disease and Associated Atherosclerosis

**DOI:** 10.1371/journal.pone.0126910

**Published:** 2015-05-20

**Authors:** Takhar Kasumov, Ling Li, Min Li, Kailash Gulshan, John P. Kirwan, Xiuli Liu, Stephen Previs, Belinda Willard, Jonathan D. Smith, Arthur McCullough

**Affiliations:** 1 Department of Gastroenterology& Hepatology, Cleveland Clinic, Cleveland, OH, United States of America; 2 Department of Research Core Services, Cleveland Clinic, Cleveland, OH, United States of America; 3 Department of Pathobiology, Cleveland Clinic, Cleveland, OH, United States of America; 4 Department of Cellular & Molecular Medicine, Cleveland Clinic, Cleveland, OH, United States of America; 5 Department of Anatomic Pathology, Cleveland Clinic, Cleveland, OH, United States of America; 6 Department of Nutrition & Medicine, Case Western Reserve University School of Medicine Cleveland Clinic, Cleveland, OH, United States of America; Northeast Ohio Medical University, UNITED STATES

## Abstract

Cardiovascular disease (CVD) is a serious comorbidity in nonalcoholic fatty liver disease (NAFLD). Since plasma ceramides are increased in NAFLD and sphingomyelin, a ceramide metabolite, is an independent risk factor for CVD, the role of ceramides in dyslipidemia was assessed using LDLR-/- mice, a diet-induced model of NAFLD and atherosclerosis. Mice were fed a standard or Western diet (WD), with or without myriocin, an inhibitor of ceramide synthesis. Hepatic and plasma ceramides were profiled and lipid and lipoprotein kinetics were quantified. Hepatic and intestinal expression of genes and proteins involved in insulin, lipid and lipoprotein metabolism were also determined. WD caused hepatic oxidative stress, inflammation, apoptosis, increased hepatic long-chain ceramides associated with apoptosis (C16 and C18) and decreased very-long-chain ceramide C24 involved in insulin signaling. The plasma ratio of ApoB/ApoA1 (proteins of VLDL/LDL and HDL) was increased 2-fold due to increased ApoB production. Myriocin reduced hepatic and plasma ceramides and sphingomyelin, and decreased atherosclerosis, hepatic steatosis, fibrosis, and apoptosis without any effect on oxidative stress. These changes were associated with decreased lipogenesis, ApoB production and increased HDL turnover. Thus, modulation of ceramide synthesis may lead to the development of novel strategies for the treatment of both NAFLD and its associated atherosclerosis.

## Introduction

NAFLD can progress from steatosis alone to non-alcoholic steatohepatitis (NASH) and ultimately to, cirrhosis. Despite these adverse hepatic outcomes, cardiovascular disease (CVD) is the major cause of death in these patients. NAFLD and its associated inflammation and fibrosis are independent predictors of the occurrence and progression of atherosclerosis [1,2]. However, it is unclear whether NAFLD is merely a marker of CVD risk, or a mediator that promotes a proatherogenic and inflammatory state.

The pathogenesis of NAFLD is largely attributed to insulin resistance induced dyslipidemia and hypercholesterolemia, which are also common features of atherosclerosis. In NAFLD, chronic insulin resistance stimulates the overproduction of triglyceride-rich VLDL, which alters HDL composition through the actions of cholesterol ester transfer protein and hepatic lipase (HL) that leads to formation of small dense HDL particles prone to degradation [3]. Thus, a defect in VLDL metabolism initiates higher levels of remnant particles and lower levels of HDL-cholesterol (HDLc), which are risk factors of atherosclerosis [4]. The total amount of atherogenic lipoproteins is reflected by circulating levels of ApoB, the major structural protein of VLDL, remnant intermediary LDL, and LDL. In contrast to VLDL and LDL, HDL and its essential protein, ApoAI, protect against atherosclerosis through a process called “reverse cholesterol transport” (RCT), and their anti-oxidant and anti-inflammatory functions [5]. Consequently, the ApoB/ApoAI ratio is considered a stronger predictor of CVD than triglycerides and cholesterol [6].

Ceramide and ceramide-derived sphingolipids are structural components of membranes, and have been linked to insulin resistance, oxidative stress, and inflammation [7–9] suggesting they may play a role in the development of NAFLD [10]. *De novo* ceramide synthesis is stimulated by inflammation and an oversupply of saturated fatty acids. Recently we demonstrated that plasma ceramides are increased in patients with NAFLD and type 2 diabetes, two conditions associated with insulin resistance [11,12]. Mechanistically, ceramide inhibits several mediators of the insulin signaling pathway including insulin receptor substrate 1 (IRS1), phosphatidylinositol 3-kinase (PI-3K) and Akt/PKB [13].

Ceramide and sphingomyelin (SM, a ceramide metabolite and precursor) are both independent risk factors of atherosclerosis [14]. Myriocin, a potent inhibitor of serine-palmitoyl transferase (SPT) [15], the rate limiting enzyme of ceramide biosynthesis, prevents atherosclerosis in ApoE knockout mice [16,17]. Myriocin also increases HDLc levels via enhanced expression of ApoAI and lecithin-cholesterol acyltransferase; proteins involved in HDL biogenesis and maturation [18].

We have demonstrated that myriocin induced reduction of ceramide production was associated with increased macrophage-specific RCT and HDL turnover, suggesting that ceramide is involved in the regulation of HDL functionality [19]. We have also shown that the decrease in plasma ceramides in morbidly obese patients after bariatric surgery coincided with decreased ApoB levels, a reduced ApoB/ApoA1 ratio and a reduction in the overall CVD risk, suggesting that in addition to regulating HDL metabolism sphingolipids may also contribute to atherosclerosis through their effect on ApoB metabolism [20]. However, the mechanism(s) linking insulin resistance-associated hepatic ceramide production to dyslipidemia and atherosclerosis in NAFLD is unknown.

This study tested the hypothesis that increased hepatic ceramide and sphingolipid production contributes to the pathogenesis of diet induced NAFLD and atherosclerosis through modulation of lipogenesis and ApoB and ApoAI metabolism. To study these questions, we used low density lipoprotein receptor deficient (LDLR^-/-^) mice, an established diet-induced model of NAFLD and atherosclerosis [21,22]. In contrast to wild type mice, the LDLR^-/-^ mouse is susceptible to diet-induced hepatic inflammation and/or fibrosis, conditions that are required for progression of simple steatosis to NASH, and development of atherosclerosis [23]. We modulated ceramide and lipoprotein metabolism through a high fat diet containing cholesterol, i.e. a Western type diet (WD) and pharmacological inhibition of sphingolipid biosynthesis. We used a heavy water (^2^H_2_O)-based metabolic labeling approach to quantify fatty acid, cholesterol, ApoAI and ApoB fluxes. To obtain mechanistic insight into the role of sphingolipids in NAFLD and associated atherosclerosis, we also assessed the expression of intestinal and hepatic genes involved in lipid and lipoprotein metabolism.

## Materials and Methods

### Materials

HPLC grade solvents for nanoflow chromatography and sample preparation were purchased from Fluka (Milwaukee, MO). [^2^H_6_]cholesterol, N-(9-fluoronylmethyloxy-carbonyl-[^13^C_6_]-leucine (L-[^13^C_6_]-Fmoc-leucine) and L-[^2^H_10_]-Fmoc-leucine, were obtained from Cambridge Isotope Laboratories, (Andover, MA), purity > 98%). DAOS (N-ethyl-N-(2-hydroxy-3-sulfopropyl)-3,5-dimethoxyaniline, sodium salt) was purchased from Dojindo Molecular Technologies (Rockville, Maryland). All other chemicals, including myriocin were from Sigma-Aldrich (St. Louis, MO). The high fat diet containing cholesterol (45% kcal lard and 0.2% cholesterol) without and with myriocin (2.2 mg myriocin/kg diet) were prepared by Teklad Diets (Harlan Laboratories, D11092803).

The labeled peptide VAPL(^13^C_6_)GAEL(^13^C_6_)QESAR for quantification of mouse ApoAI was synthesized as described [19]. The total mouse ApoB (ApoB48 + ApoB100) in plasma was quantified based on endogenous LSVDQFVR peptide that represent in full length (ApoB100) and the truncated protein (ApoB48) using the synthetic labeled peptide L(^2^H_10_)SVDQFVR as an internal standard. L(^2^H_10_)SVDQFVR was synthesized using a solid-phase method in the Cleveland Clinic Molecular Biotechnology Core on a 396 52 Peptide Synthesizer (Advanced Chem Tech, Louisville, Kentucky). Stable isotope labeled L-[^2^H_10_]-Fmoc-leucine was coupled in the peptide sequence to give a molecular mass shift of 10 Da from the unlabeled endogenous peptide. The molecular weight of the purified peptide was verified by ESI-MS and was found to yield [M + 2H]^+2^ ion with the expected 487.3 M/z and an isotopic purity of 97%. The stock solution of labeled peptides VAPL(^6^C_13_)GAEL(^6^C_13_)QESAR and L(^2^H_10_)SVDQFVR were standardized using an HPLC-UV based amino acid analysis performed in the Dr. John Crabb’s lab in Cole Eye Institute, Cleveland Clinic. The working solutions made at a concentration of 10 and1 μM for ApoAI and ApoB, respectively (pH = 3). These solutions were divided into 0.05 ml fractions and stored at -80°C until usage.

### Animal studies

All experiments were performed in accordance with protocols approved by the Cleveland Clinic Institutional Animal Care and Use Committee (ICUC). Studies were performed in LDLR^-/-^ mice, an established model of diet-induced insulin resistance, NAFLD, and atherosclerosis.

Eight- to 10-week-old male LDLR^-/-^ mice housed in our animal care facility with a 12:12 h light: dark cycle, had free access to food and water. Mice were randomized into three groups and fed an additional 12 weeks with a standard chow diet (SD, 20% kcal protein, 70% kcal carbohydrate and 10% kcal fat, Harlan Teklad)), a high saturated fat and cholesterol-containing diet (WD, TD88137, Harlan Teklad, Madison, WI, 45% kcal from fat, containing 21% of fat—saturated fat/total fat ratio = 0.64 and 0.2% cholesterol) or WD plus myriocin (2.2 mg/kg diet). Composition of high fat diet containing cholesterol is presented in **S1 Table**. Food intake was measured twice a week and body weight was recorded weekly. In contrast to wild-type mice, a high fat diet containing cholesterol (0.15–0.25% w/w) results in hepatic inflammation and atherosclerosis in LDLR^-/-^ mice after 12 weeks [24,25]. A Western diet also increases hepatic SPT activity, ceramide and SM levels in these mice [26]. Similar amounts of cholesterol in the low fat diet induces only modest atherosclerosis in these animals [27], without causing insulin resistance and NAFLD [28].

#### Glucose and Insulin Tolerance Tests

Glucose and insulin tolerance tests were performed at the end of the diet experiment before the tracer study. The glucose tolerance test was initiated after 6 hours of fasting. After the baseline measurement of glucose from tail vein blood, a glucose solution in distilled water (20% w/w, 1 mg/g body weight) was administered by intraperitoneal injection. Glucose tolerance was assessed from blood glucose measures obtained at 15, 30, 60 and 120 min after glucose administration.

The insulin tolerance was assessed in fed mice by measuring blood glucose levels prior to and 15, 30, 45 and 60 min after intraperitoneal human insulin (Humulin; Lilly; 0.75 units/ kg body weight) injection (0.75 units/kg body weight). Insulin sensitivity and glucose utilization were calculated based on the area under the blood glucose response curve.

#### Hepatic Lipogenesis, HDL and ApoB Turnover

Hepatic fatty acid and cholesterol synthesis were assessed based on ^2^H-incorporation into palmitate and cholesterol [29]. HDLc, ApoAI and ApoB turnover were quantified as previously described [19,30]. Briefly, two days after taking baseline blood samples through bleeding from the lateral saphenous vein, LDLR^-/-^ mice (n = 6 in each group) received a loading dose of ^2^H_2_O saline solution (20 μl/g body weight by Intraperitoneal injection) and were given drinking water enriched with ^2^H_2_O (5%). Saphenous vein blood samples (~80 μl) were collected at 4, 8, 24 and 72 hrs. After seven days of ^2^H_2_O exposure, animals were sacrificed following the terminal blood sample (~1ml) collection by cardiac puncture. The heart was perfused with cold PBS. The heart and a small fraction of the liver were saved in formalin solution for future histological analysis. The remaining part of the liver and the intestine were freeze clamped in liquid nitrogen, and saved at -80°C. Blood samples were immediately centrifuged (2000 g for 10 minutes) and plasma was isolated. Plasma was used for the analysis of ^2^H-labeling of the total body water (3 μl), ApoAI and ApoB (5 μl), and for the isolation of the HDLc fraction (25 μl), as described below.

#### Triglyceride and ApoB Secretion

Triglyceride secretion rates were measured in 6 h fasted mice using the Tyloxapol method [31]. After collecting the baseline blood sample, 1g/kg of Tyloxapol solution (0.15 g/ml in 0.9% saline) was intra-peritoneally injected to each mouse. Tyloxopol prevents triglyceride hydrolysis and clearance. Additional blood samples (80 μl) were collected at 30 and 60 min through the saphenous vein. Mice were sacrificed at 120 min post-injection and the terminal blood samples were collected by cardiac puncture. Triglyceride secretion was assessed based on plasma triglyceride concentrations measured at different time points.

### Analytical Procedures

Aspartate aminotransferase (AST) and alanine aminotransferase (ALT) were measured using commercial kits (Diagnostic Chemicals, Ltd., Oxford, CT). Plasma and hepatic triglyceride levels were determined by using Triglyceride GPO reagent (Pointe Scientific, Lincoln Park, MI). Hepatic and plasma total cholesterol, plasma HDLc (in ApoB-depleted plasma), and hepatic total palmitate levels were measured by GC-MS [19]. Blood glucose was monitored using the OneTouch Ultra Blood Glucose Meter and test strips (Lifescan, Milpitas, CA) and plasma insulin levels were measured using an ELISA assay (Mercodia, Uppsala, Sweden).

#### Sphingolipids: Ceramide Analysis by Mass Spectrometry

Ceramide species were quantified in liver and plasma as previously described [32]. Briefly, an aliquot of powdered liver tissue (25–35 mg) was suspended in ice-cold saline (500 μl, 1M NaCl) and spiked with C17:0 ceramide internal standard. Lipids were extracted and ceramide subspecies analyzed by HPLC coupled with on-line electrospray ionization tandem mass spectrometry. Total ceramide level was calculated from the sum of analyzed ceramide species. A similar protocol was used to analyze plasma ceramides, except that after lipid extraction, ceramides were semi-purified using silica gel loaded column chromatography.

#### SM Analysis by UV Spectroscopy

SM levels in plasma and liver samples were determined as described [17]. Briefly, SM was hydrolyzed by bacterial SMase and converted to N-acylsphingosine and phosphorylcholine. Subsequently, alkaline phosphatase was used to convert phosphorylcholine to choline, which was then oxidized by choline oxidase to generate H_2_O_2_. Liberated H_2_O_2_ was detected using DAOS (N-ethyl-N-(2-hydroxy-3-sulfopropyl)-3,5-dimethoxyaniline, sodium salt) and 4-AAP (4-aminoantipyrine) and horseradish peroxidase to generate a blue colored dye. Samples were run in parallel with the calibration curve samples of SM. Absorption was measured at 600 nm using a plate spectrophotometer. The amount of SM in biological samples was calculated by comparing it to a standard curve of SM.

#### Gene Expression Analyses

Total RNA was isolated from the mouse liver tissue using RNeasy Mini Kit (Qiagen Inc. Valencia, CA). One μg of total RNA was reverse transcribed using the commercial Advantage RT-for-PCR Kit (Clontech Laboratories, Inc. Mountain View, CA) with random decamers as primer. Real-time PCR amplification was performed using Brilliant SYBR Green QPCR Master Mix (Agilent Technologies, Inc. Santa Clara CA) and gene-specific primers in an Mx3000p PCR machine (Agilent Technologies, Inc. Santa Clara CA) in duplicate. The relative amount of target mRNA was determined using the cycle threshold (Ct) method by normalizing target mRNA Ct values to that of β-actin. Fold induction ratios were calculated relative to basal conditions for each group using the formula: 2^−ΔΔCt^. The list of primer sequences used for qRT-PCR is presented in **S2 Table**. All primers used for real-time PCR analysis were synthesized by Integrated DNA Technologies, Inc (Coralville, NJ).

#### Western Blot Analysis

Proteins for each sample (20 μg) were resolved on 4–20% pre-cast gradient SDS-PAGE gels (Life Technologies, NY), and then electro-transferred to polyvinylidene difluoride membranes. The membranes were incubated with primary antibodies overnight in 4°C, followed by horseradish peroxidase-labeled second antibody (Santa Cruz Biotech, CA) for 1 h at room temperature. ImageJ software (NIH) was used to measure band intensities. Primary antibodies were—Anti-Actin (Santa Cruz Biotech, CA); Anti-SRB1 (Pierce Biotech, IL), anti-Akt, anti-Phosho-Akt, anti-PI3K, and anti- ABCA1 (Millipore, CA). Finally, detection procedures were performed using an ECL Plus Western Blotting Detection Kit (Amersham, Piscataway, NJ).

#### Hepatic Oxidative Stress

The hepatic oxidative stress markers, reduced glutathione (GSH) and dithiothreitol (DTT)-reducible oxidized glutathione (GSSG) were analyzed as described [33]. Briefly, liver tissue samples (~50 mg) were spiked with 10 μL of *homo*-glutathione (20 μg/mL) internal standard and extracted with 200 μL of water using a Polytron homogenizer. The proteins were precipitated by adding 800 μL of acetonitrile and centrifugation. To derivatize reduced glutathione, the supernatant was incubated with 0.1 mL of 50 mM iodoacetamide for 45 min. Solvents were evaporated to dryness under nitrogen at 50°C for 20 min in a TurboVap LV (Caliper LifeSciences, Inc, USA). The residue was reconstituted in 100 μL of formic acid in water (0.1%): acetonitrile (99:1). Five μL of this solution was injected onto the HPLC-MS system. Ions with m/z 365.1 and 379.1 were analyzed for quantification of glutathione and homoglutathione abundance.

For the analysis of total glutathione, 0.2 mL of DTT (0.1 M) was added to the tissue extract to reduce oxidized glutathione. After 15 min, 0.2 mL of iodoacetamide (0.1 M) was added and samples were analyzed as above. Oxidized glutathione was quantified based on differences in total and reduced glutathione levels.

#### Hepatic Palmitate and Cholesterol Analysis

^2^H-enrichment of body water was measured using a modified acetone exchange method [34]. To quantify hepatic lipogenesis, liver samples (~30–40 mg) in parallel with the calibration curve samples were spiked with 50 μL of 1 mM [^2^H_6_]-cholesterol and 100 μL of 1mM heptadecanoic acid (C17) solutions, homogenized in 0.5 ml of 1N NaCl and extracted by the Bligh-Dyer method [35]. After the evaporation of the solvents, the lipids were saponified with 1N KOH/70% ethanol (v/v) for 2 hours at 70°C, vortexing occasionally. Samples were then evaporated to dryness and suspended in 150 μl of 1N HCl. Total lipids were extracted with 1.0 ml of pentane. The solvent was evaporated and the dried residue was derivatized with 65 μl of bis(trimethlsilyl) trifluoroacetamide + 1% trimethylchlorosilane (TMS) at 70°C for 45 min. The ^2^H enrichment and concentration of cholesterol and palmitate were determined using an Agilent 5973N-MSD equipped with the Agilent 6890 GC system. Cholesterol was analyzed in Electron impact ionization (70 eV) mode with SIM of m/z 368–371 (M_0_-M_3_, endogenous cholesterol) and 374 (M_6_, [^2^H_6_]-cholesterol internal standard). Palmitate was analyzed with SIM of m/z 313–316 (M_0_-M_3_, endogenous palmitate) and 327 (M_0_-heptadecanoate internal standard). Hepatic cholesterol and palmitate contents (nmol/gram wet weight) were quantified using a calibration curve and the ratios of the integrated peak areas.

#### HDLc Analysis

ApoB-depleted plasma samples for the analysis of HDLc were prepared from 25 μL of plasma using a magnesium chloride/dextran sulfate reagent (Stanbio Laboratory, Boerne, TX)[19]. The supernatant containing the ApoB-depleted plasma was recovered and used for the analysis of HDLc. Plasma HDLc levels were quantified using the isotope dilution method by mass spectrometry. Briefly, the ApoB-depleted plasma samples were spiked with 50 μL of 1 mM [^2^H_6_]-cholesterol solution in chloroform. HDL proteins were precipitated with 1 mL of cold acetone (-20°C). Samples were incubated at -20°C for 4 hours and then they were centrifuged at 2000 g for 5 minutes. The supernatant was used for the analysis of HDLc. After the evaporation of acetone, total HDLc was analyzed by the GC-MS as described above.

#### ApoAI and ApoB Analysis

ApoAI and ApoB were analyzed using a proteomics approach as described [30]. Briefly, proteins from 5 μl of plasma were precipitated using 800 μL of cold acetone (-20°C). Pellets were washed with 600 μL of cold acetone and centrifuged at 2000 g for 5 minutes. The supernatants were discarded and the pellets were air-dried. Proteins were dissolved in 360 μL of 0.1 M ammonium bicarbonate solution pre-mixed with 40 μL of 10% sodium deoxycholate. After incubating samples for one hour at room temperature, 20 μL of this solution was spiked with stable isotope labeled synthetic peptide solutions: L(^2^H_10_)SVDQFVR (10 pmol) for ApoB and VAPL(^13^C_6_)GAEL(^13^C_6_)QESAR (100 pmol) for ApoAI. To reduce the cysteine residues, the samples were reacted with DTT solution (2.5 μL of 0.2 M) in Tris buffer (100 mM pH 8) for 20 minutes at room temperature. Samples were alkylated with an excess of iodoacetamide (9 μL of 0.2M) for 20 minutes at room temperature. The excess of iodoacetamide was reacted with DTT (8 μL of 0.2 M). The proteins were completely digested in solution with the addition of Promega sequencing grade trypsin (10 μL of 100 ng/μL trypsin solution in 100 mM pH 8 Tris buffer) at room temperature overnight. Samples were desalted through solid phase extraction using a Pierce C18 Pepclean spin column. The peptides were eluted with 2 X 20 μL of 70% acetonitrile and the solvent was evaporated in a Speedvac. Samples were reconstituted in 30 μL of 1% acetic acid. Five μL of the sample solution was injected for the LC-MS analysis.

#### LC-MS/MS Analysis of ApoAI and ApoB

The chromatographic separation of the protein digest was performed by a Dionex ultimate 3000 HPLC (Thermo Scientific). Samples were first loaded on an Acclaim PepMap100 Nano-Trap Column (100μm x 2cm, C18, 5 μm, 100Å, Thermo Fisher Scientific), and then separated by an Acclaim PepMap RSLC reverse phase nano-column (75 μm x 15 cm, C18, 2 μm, 100Å, Thermo Fisher Scientific) with a mobile phase A (0.1% formic acid in water) and B (5% water in acetonitrile with 0.1% formic acid). A 140-minute stepwise gradient was started with 2% of mobile phase B. After 10 minutes of desalting, mobile phase B was linearly increased to 40% in 100 minutes. Mobile phase B was then ramped to 80% in 5 minutes and then held at 80% B for 15 minutes. Subsequently mobile phase B was decreased to 2% in 2 minutes and equilibrated for 13 minutes with 2% of B. Tandem mass spectra were recorded on a Thermo LTQ Orbitrap Elite (Thermo Electron Corp., Bremen, Germany) and operated in a positive ion mode using electro spray ionization (ESI). The peptides were infused at a flow rate of 300 nL/min via a noncoated PicoTip emitter (FS360-75-15-N-512, New Objective Inc., Woburn, MA) at a spay voltage of 2.2 kV. The inlet capillary temperature was maintained at 175°C. A data-dependent method was used for data acquisition. Each full MS scan using Orbitrap (high resolution FT instrument) at 60,000 resolution at m/z 400 was followed by 20 collisionally induced dissociation (CID) MS/MS scans on a LTQ Velos pro mass spectrometer.

#### Database Search for the Identification of ApoAI and ApoB

For the identification of proteins, including ApoAI and ApoB, the MS data were analyzed using all CID spectra collected in the experiment. Peak lists were generated using Thermo Electron Proteome Discoverer V1.3 software and compiled into Mascot generic format (.mgf). The. mgf files were searched against the National Center for Biotechnology Information *mouse* reference sequence database (ftp://ftp.ncbi.nih.gov/refseq/) released on December 20^th^, 2011 containing 30,438 entries. The search was performed using carbamidomethyl as a fixed modification of cysteine, oxidation as an optional modification of methionine and allowing one missed cleavage. The mass tolerances for the precursor and product ions were 15 ppm and 1.5 Da, respectively. A score of >35 was considered as significant. The interpretation process was aided by additional searches using Blast (http://blast.ncbi.nlm.nih.gov/Blast.cgi) as needed. Mouse ApoAI and ApoB were identified based on multiple unique peptides with 99% confidence.

#### Calculations

^2^H-isotope incorporation into ApoAI and ApoB were assessed based on the mass isotopomer distribution analysis of their tryptic peptides by the high resolution full scan spectra recorded on an Orbitrap MS. Mass isotopomers are molecules that differ by the presence of different heavy isotopes resulting in a mass spectrum with a monoisotopic peak (M0) followed by distinct heavy isotopomer (Mi, where i is an integer >0) peaks. The quantification was performed by integrating each isotopomer of a given chromatographic peak within a defined mass range (Mi±0.1 m/z).

The molar percent enrichment of an isotopomer (MPE M_i_) was calculated as:

MPEofMi=AMi/sum(AM0+AM1+⋯+AMn) (1)

where, AM_i_ represents the area under the curve for i^th^ isotopomer.

Total labeling was calculated using the formula:

MPE=MPEM1×1+MPEM2×2+⋯+MPEMi (2)

Net labeling (isotopic excess) due to ^2^H-incorporation is calculated as the difference of total MPE at each time point and the baseline MPE calculated at t = 0.

Unique peptides VAPLGAELQESAR (analyzed as [M+2H]^+2^ ion with m/z of 670.87) and LSVDQFVR (analyzed as [M+2H]^+2^ ion with m/z of 482.27) were used for the analysis of ApoAI and ApoB, respectively. The fractional catabolic rates (FCRs) were determined based on a single compartment model by fitting a time course of M_1_ and net labeling of analytes, to an exponential rise curve equation:

$$E(t)=E0\;\times \left(1-e^{-kt}\right)$$ (3)

At steady state, the rate constant represents both the fractional synthesis rate (FSR) and the fractional catabolic rate (FCR). Since in the WD group hepatic ^2^H-labeling of cholesterol is very low and it linearly increases during 7 days of ^2^H_2_O exposure, FSR of total cholesterol was calculated using the formula:

FSR=MPEofHepaticCholesterol/(N×MPEofplasmawater×time) (4)

where MPE is net labeling of hepatic cholesterol at the 7 days of ^2^H_2_O administration. N represents the number of exchanged hydrogen atoms and assumed to be 26 for cholesterol [36].

The production rates (PR) of ApoB, ApoAI and HDLc were calculated as the product of FCR and their respective pool size:

$$PR\;\left(\frac{g}{kg\times h}\right)=pool\;size\;\times FCR$$ (5)

where the pool size (absolute content) in circulation is the product of HDLc, ApoAI, or ApoB concentrations and plasma volume, estimated as 45 ml/kg body weight. The plasma levels of mouse ApoAI were quantified using the ratios of the integrated peak areas of the endogenous mouse ApoAI peptide VAPLGAELQESAR to the heavy labeled synthetic peptide VAPL(^13^C_6_)GAEL(^13^C_6_)QESAR (670.86/676.88) from the full scans. The plasma ApoB level was quantified using the ratio of the integrated peak areas of the endogenous mouse ApoB peptide LSVDQFVR to the heavy labeled synthetic peptide L(^2^H_10_)SVDQFVR (482.27/487.29) from the full scans.

Similar calculations were performed for the PR of both hepatic cholesterol and palmitate. The pool sizes of hepatic cholesterol and palmitate (mg/ per g liver wet weight) were calculated using the isotope dilution method by GC-MS.

#### Liver Histology and Immunohistochemistry

Formalin-fixed paraffin-embedded livers were sectioned and stained with hematoxylin and eosin (H&E) and Masson’s trichrome. Liver histology was evaluated in a blinded manner by an experienced pathologist (X. Liu). Apoptosis was assessed using the TUNEL assay (ApopTag Peroxidase in Situ Apoptosis Detection Kit, Millipore). TUNEL was visualized using the ApopTag plus In Situ Apoptosis Detection kit (S7101; Millipore, CA). Image-Pro Plus software (Media Cybernetics, MD) quantified apoptosis by counting the number of TUNEL-positive cells in 30 random microscopic fields (20x).

#### Aortic Root Lesion Quantification

Aortic roots were fixed in phosphate-buffered formalin and sectioned. Processing, staining and lesion quantification were carried out as described [37].

#### Statistical Analysis

The average duplicate of GC-MS and LC-MS injections, which differed less than 2%, were used for mass-spectrometric analysis. Six mice were studied at each time point unless indicated otherwise. All data were presented as mean±SEM. Differences between groups were tested using one-way ANOVA followed by Tukey’s *post hoc* procedure for multiple comparisons. *P*<0.05 was considered statistically significant.

## Results and Discussion

### Results

#### Effect of diet and inhibition of ceramide on weight gain and insulin sensitivity

**Fig 1:**
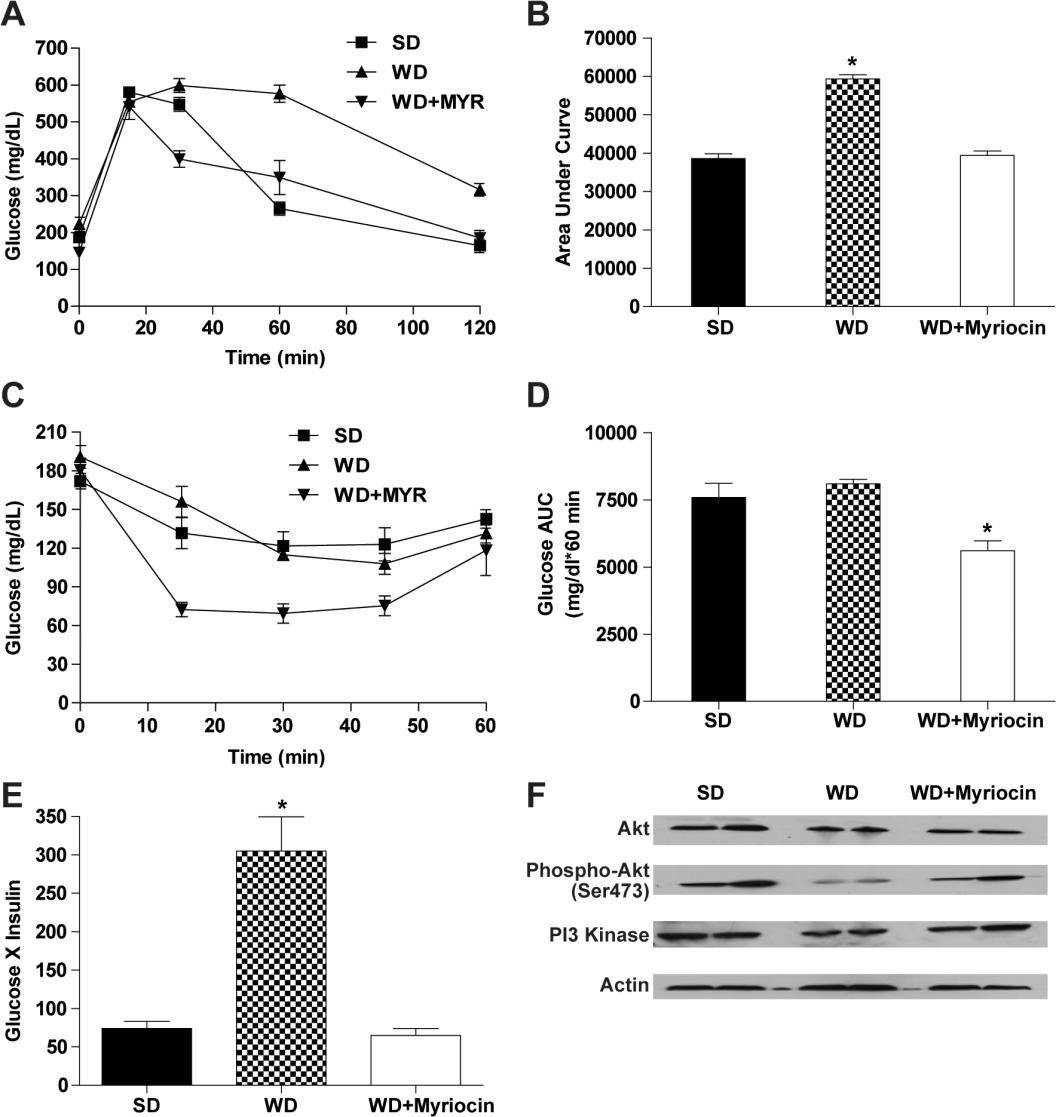
Myriocin improves glucose utilization and insulin sensitivity in LDLR^-/-^ mice (mean ± SEM, n = 6). Glucose levels (**A**) and area under the curve during a glucose tolerance test obtained at 0, 15, 30, 60, and 120 min (**B**). Glucose levels (**C**) and area under the curve during the insulin tolerance test was measured at 0, 15, 30, and 60 min post-injection (**D**). Index of insulin sensitivity (insulin x glucose) in fasted mice (**E**). Hepatic expression of PI 3-Kinase, Akt and phospho-Akt (serine 473) in LDLR^**-/-**^ mice fed a SD, WD, or a WD + myriocin (**F**). *P<0.05, significantly different from all other groups.

The metabolic effects of a WD and myriocin were evaluated in LDLR^-/-^ mice fed a WD with and without myriocin. As shown in **Table 1**, WD fed LDLR^-/-^ mice weighed more but there were no differences in food intake among the groups. Myriocin significantly reduced the weight gain in the WD group. The high fasting glucose and insulin levels (**Table 1)** and abnormal glucose tolerance test (**Fig 1A and 1B**) caused by the WD were normalized by myriocin.

**Table 1:** Effect of a Western diet and myriocin on body weight, food intake and fasting plasma biochemistries in LDLR^-/-^ mice.

	SD	WD	WD + myriocin
**Body weight (g)**	23.7±1.0	39.3±1.2^a^	29.4±0.9^b^
**Food Intake (g/day)**	2.2±0.1	2.3±0.1	2.2±0.1
**Glucose (mg/dL)**	180.8±2.0	224.6±14.3^a^	146.0±8.5^a^
**Insulin (ng/mL)**	0.54±0.04	1.43±0.06^a^	0.63±0.05
**Cholesterol (mg/dL)**	121.4±9.7	758.8±44.8^a^	201.5±11.4^b^
**HDLc (mg/dL)**	69.5±3.6	47.4±5.1^a^	68.9±6.9^c^
**TGs (mg/dL)**	201.3±9.0	691.2±67.4^a^	162.7±10.4^a^
**ApoB (mg/dL)**	70.7±4.8	234.4±23.7^a^	108.7±13.6^a^
**ApoAI (mg/dL)**	219.4±4.2	222.7±8.5	298.8±9.9^a^
**ApoB/ApoAI**	0.32±0.02	1.04±0.08^a^	0.37±0.05

^a^ *P* <0.05, different from two other groups

^b^ *P* <0.05, different from the SD group

^c^ *P* <0.05, different from the WD group.

Values represent mean ± SEM (n = 6).

Although the insulin tolerance test did not differ between SD and WD groups **(Fig 1C and 1D),** the insulin sensitivity index (the product of fasting insulin and glucose levels) [38] was almost 4 fold higher with the WD (**Fig 1E**). Myriocin improved insulin sensitivity; glucose tolerance, and the insulin sensitivity index (**Fig 1B, 1D and 1E**). These observations demonstrate that myriocin prevents WD-induced obesity and systematic insulin resistance in LDLR^-/-^ mice.

To determine whether the insulin sensing effect of myriocin took place in the liver, we examined the protein levels of PI3K and its product phosphor-Akt in the liver. The WD reduced expression of both PI3K and its product phosphor-Akt, which reflects hepatic insulin resistance, was reversed by myriocin treatment (**Fig 1F**). Thus, pharmacological inhibition of ceramide biosynthesis with myriocin improves diet-induced hepatic insulin resistance.

#### Effect of diet and inhibition of ceramide on hepatic and plasma ceramide and SM levels

**Fig 2:**
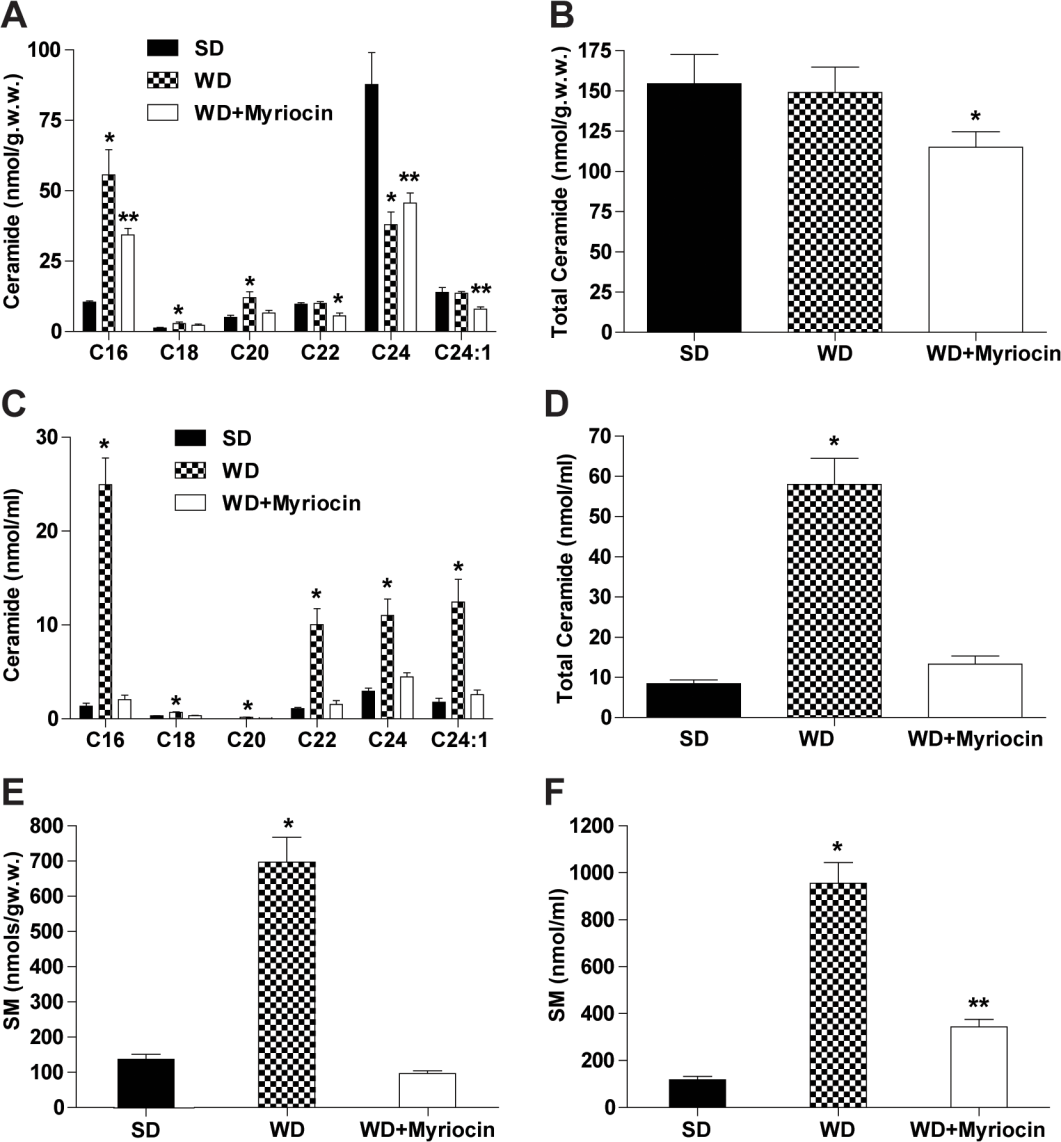
Myriocin reduces hepatic and plasma ceramides and sphingomyelin (SM) in LDLR^-/-^ mice (mean±SD, n = 6). Distribution of ceramide species in the liver (**A**). Total amount of ceramides in the liver (**B**). Distribution of ceramide species in plasma (**C**). Total ceramides in plasma (**D**), Hepatic (**E**) and Plasma (**F**) levels of SM. *P<0.001, compared to the SD and WD + myriocin groups. **P<0.01, compared to the WD group.

To determine the role of sphingolipids on diet-induced NAFLD and atherosclerosis, we quantified hepatic and plasma levels of different ceramide species and total SM. The targeted lipidomics approach revealed that a WD increased hepatic levels of C16:0, C18:0 and C20:0 ceramides, while no differences were observed in C22:0 and C24:1 ceramides. In contrast, C24:0 was reduced due to a WD (**Fig 2A**). Since C24:0 is the major hepatic ceramide, its reduction counter balanced increases in the other species and resulted in a similar level of total ceramides (**Fig 2B**). Similar hepatic redistribution of ceramide species in LDLR^-/-^ mice fed a high-fat diet has been documented in a previous study [39]. Myriocin partially normalized the hepatic ceramide levels (reduced long-chain ceramides and increased C24:0) resulting in lower total ceramide levels compared to both SD and WD groups.

To assess the effect of increased dietary fatty acid supply on plasma ceramides and SM levels, we also quantified ceramides in plasma. Plasma levels of total ceramide and all ceramide species were elevated in WD (**Fig 2C and 2D**) with the major changes observed in C16:0, C22:0, C24:0 and C24:1, which were increased ~20, 4 3 and 6 fold, respectively (**Fig 2C**). Myriocin normalized both total and individual ceramides in plasma. The increased levels of hepatic SM were also normalized by myriocin (**Fig 2E**). Myriocin treatment significantly reduced plasma SM as compared to the WD group, but it did not completely eliminate WD-induced alterations (**Fig 2F**). Thus, these data indicate that systematic fatty acid oversupply increases plasma ceramides and SM levels through *de novo* synthesis that was reduced by pharmacological inhibition with myriocin.

#### Effect of diet and inhibition of ceramide on plasma lipids and lipoproteins

To assess the role of ceramides in diet-induced dyslipidemia, we also quantified plasma lipid and lipoprotein metabolism. A WD significantly increased plasma total cholesterol and triglycerides and significantly reduced HDLc levels **(Table 1)**. Myriocin reduced total plasma cholesterol and triglycerides and increased HDLc. Plasma ApoB, but not ApoAI, increased on the WD resulting in a ~4 fold increase in ApoB/ApoAI ratio. Myriocin treatment stabilized ApoB levels and significantly increased ApoAI levels resulting in a normalized ApoB/ApoA1 ratio suggesting that ceramides are involved in hepatic lipoprotein metabolism.

To determine if myriocin improved dyslipidemia by decreasing lipoprotein-bound triglyceride secretion, plasma triglycerides were measured at different time points after treatment with tyloxopol, which inhibits lipolysis. Myriocin reversed a WD induced triglyceride secretion (*P*<0.05) (**S1 Fig**). Collectively, these results suggest that ceramides regulate hepatic lipid and lipoprotein metabolism.

#### Effect of diet and inhibition of ceramide on ApoB and HDL turnover

To understand the mechanism of WD and myriocin-induced changes in lipoprotein metabolism and ApoB/ApoAI ratio, we quantified ApoB and ApoAI kinetics with ^2^H_2_O. Intraperitoneal bolus loading of ^2^H_2_O (20 μl/g body weight) followed by free access to 5% enriched ^2^H_2_O drinking water maintained body water at a steady state labeling of ~2.8–3%. Time course ^2^H-labeling of total body water in LDLR^-/-^ mice is presented in **S2 Fig.**

#### ApoB kinetics

**Fig 3:**
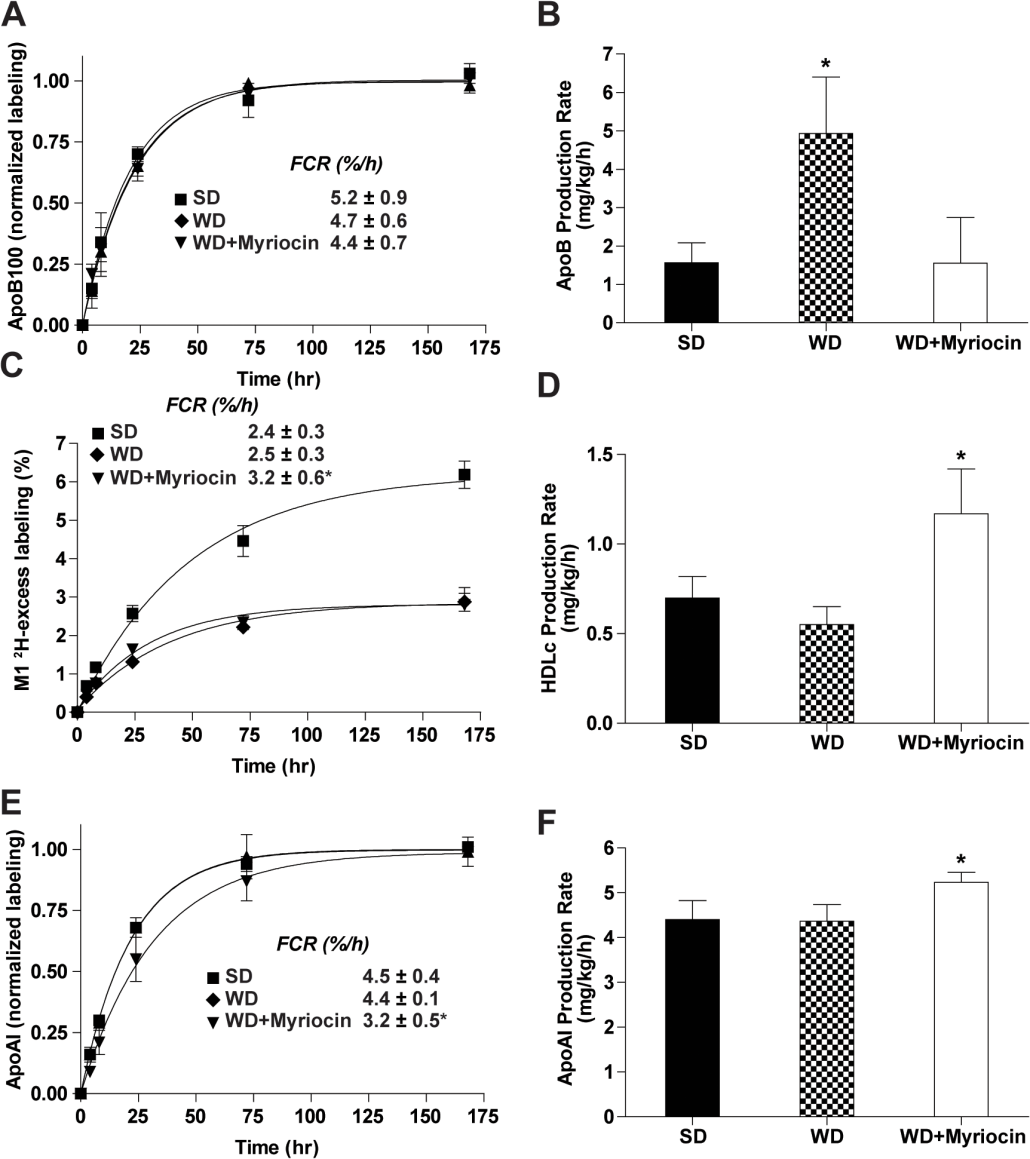
Effect of myriocin on ApoB and HDL turnover in LDLR^-/-^mice. ApoB fractional catabolic rate (FCR) (**A**) and production rate (PR) (**B)**, HDLc FCR (**C**) and PR (**D**)and ApoAI FCR (**E**) and PR (**F**). Insets give the corresponding FCR (mean ± SD, n = 5). *P<0.05 significantly different from all other groups.

As expected, the ApoB fractional catabolic rate (FCR) was much slower in LDLR^-/-^ mice (5.2±0.9%/h) as compared to rates previously reported for wild type mice (~10%/h) on a similar SD [40] **(Fig 3A)**. WD (4.7±0.6%/h) and WD+myriocin (4.4±0.7%/h) had no effect on FCR of ApoB. In contrast, the ApoB PR tripled on a WD and was completely normalized with myriocin (**Fig 3B**).

#### HDLc and ApoA1 kinetics

As shown in Fig 3C, myriocin increased the turnover of HDLc (FCR) as compared to both SD and WD (3.2±0.6%/h vs 2.4±0.3%/h and 2.5±0.3%/h)) (**Fig 3C**), and increased the PR of HDLc (1.20±0.32 mg/kg/h in WD+myriocin vs 0.70±0.12 and 0.59±0.11 mg/kg/hr in SD and WD, respectively, *P*<0.005) (**Fig 3D**). Myriocin treatment increased the PR and decreased the FCR of ApoAI compared to both the SD and WD groups (**Fig 3E and 3F**). Thus, although a WD had no effect on HDL turnover, myriocin treatment increased both HDLc and ApoAI flux, suggesting increased flux of cholesterol in the RCT pathway.

**Fig 4:**
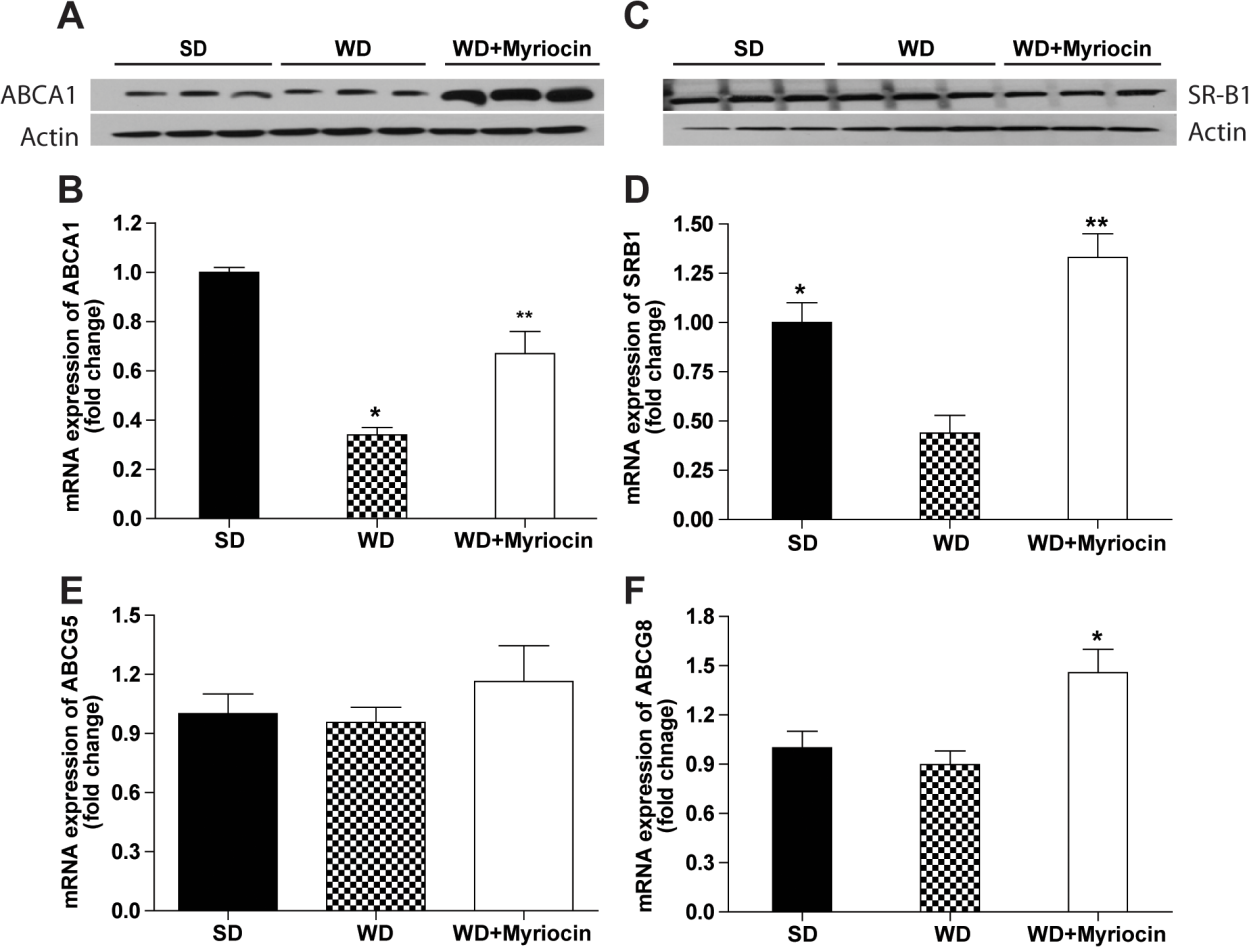
The effect of myriocin on hepatic expression of genes and proteins involved in reverse cholesterol transport in LDLR^-/-^ mice (mean ± SEM, n = 6). Protein expression and mRNA of hepatic ABCA1 (**A, B**) and SR-B1 (**C, D**). mRNA expression of hepatic ABCG5 and ABCG8 (**E** and **F).** Quantitative real-time-PCR was normalized relative to 18S. *P<0.05, compared to the SD group. **P<0.05, compared to the WD group.

To determine if myriocin’s effect on HDL flux was related to changes in RCT, we measured the hepatic expression of genes and proteins involved in key steps of RCT. WD reduced the expression of ATP-binding cassette transporter A1 (ABCA1) mRNA and protein, the major receptor involved in hepatic HDL biogenesis (**Fig 4A and 4B**). This was associated with a 3-fold reduction of scavenger receptor type BI (SR-B1) mRNA involved in selective hepatic uptake of HDL (**Fig 4D**). However, no differences were observed in SR-B1 protein (**Fig 4C**) suggesting post-transcriptional regulation of this protein. Myriocin treatment increased ABCA1 protein levels and reversed WD-induced changes in ABCA1 and SR-B1 gene expression. The WD produced no significant changes in the expression of hepatic mRNA levels of ATP-binding cassette transporters Abcg5 and Abcg8, the half-transporters involved in hepatobiliary cholesterol elimination. However, myriocin treatment caused a 45% induction of ABCG8 compared to the WD (**Fig 4F)**. Similar, but not significant increase was observed in ABCG5 mRNA expression, due to myriocin treatment (**Fig 4E).** Consistent with HDL flux results, these data indicate that myriocin increased biogenesis and selective hepatic uptake of HDL and hepatobiliary elimination of cholesterol, all suggesting increased RCT.

#### Effect of diet and inhibition of ceramide on hepatic steatosis, inflammation, oxidative stress and apoptosis

**Fig 5:**
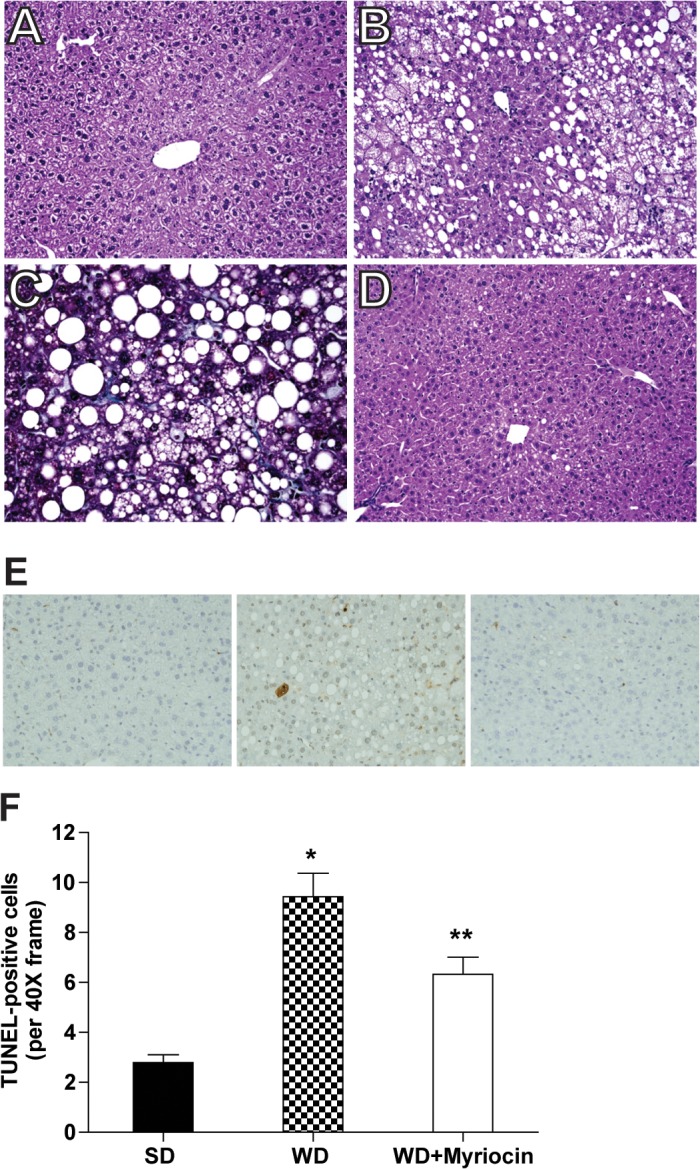
Myriocin prevents steatosis, inflammation, fibrosis and apoptosis in LDLR^-/-^ mice fed a WD. Paraffin-embedded sections of liver tissue were stained with hematoxlin and eosin (H&E) (**A, B, D**) and trichrome **(C)** and evaluated at 200X and 400X, respectively. As compared to the SD (**A),** the WD induced steatosis, lobular inflammation, mild hepatocyte ballooning (**B)** and focal pericellular fibrosis **(C)** that was ameliorated by myriocin treatment **(D)**. Representative TUNEL-stained liver sections. TUNEL-positive nuclei (brown) labeled with diaminobenzidine (DAB) were detected in the WD group **(E)** and expressed as a percent of DAB-positive nuclei (mean ± SEM, n = 6) (**F)**. *P<0.05 significantly different from the SD and WD + Myriocin groups. **P<0.05 significantly different from the SD group.

A key question is whether hepatic ceramide biosynthesis is involved in diet-induced NAFLD. As compared to SD, the WD caused hepatocyte disarray, lipid accumulation, lobular inflammation, and mild centrilobular fibrosis (**Fig 5A–5C**). These histological abnormalities were prevented by myriocin (**Fig 5D)**. There was a 10-fold increase in hepatic triglyceride levels in WD, which was reduced by 65% with myriocin (**S3 Fig).** The WD increased the number of TUNEL-positive nuclei. Myriocin treatment significantly reduced but did not eradicate this apoptotic response to the WD **(Fig 5E and 5F).**

**Fig 6:**
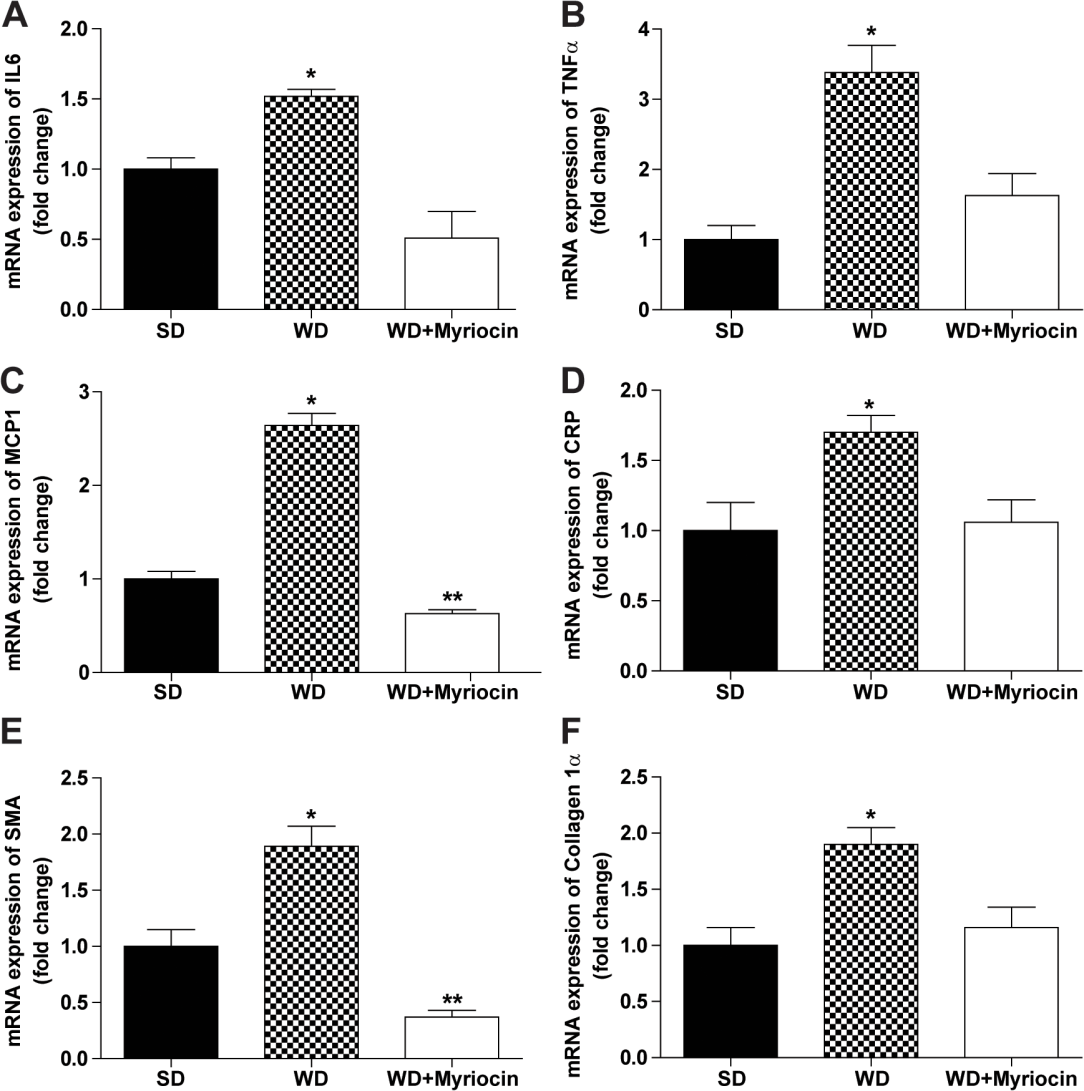
Myriocin prevents a WD-induced generation of pro-inflammatory and pro-fibrotic markers (mean ± SEM, n = 6). The mRNAs encoding interleukin 6 (IL6) (**A**), tumor necrosis factor α (TNFα) (**B**), monocyte chemoattractant protein-1 (MCP1) (**C**), C-reactive protein (CRP) (**D**), smooth muscle actin (SMA) (**E**) and collagen 1α (**F**) were analyzed by qRT-PCR relative to 18S. *P<0.05 compared to the SD and WD+Myriocin groups. **P<0.05 significantly different from the SD group.

A WD induced pro-inflammatory and pro-fibrotic mediators ascertained by the expression of IL-6, TNFα, MCP1, CRP, SMA and collagen 1α messenger RNAs, which were blunted by myriocin (**Fig 6**). A WD induced oxidative stress, as assessed by the glutathione redox ratio (GSH/GSSG). Myriocin did not attenuate a WD-induced oxidative stress in LDLR^-/-^ mice (**S4 Fig**). Despite histological alterations and hepatic oxidative stress, the WD did not alter plasma ALT and AST concentrations (data not presented).

Overall, these results show that inhibition of ceramide synthesis is associated with a profound reversal of diet-induced hepatic steatosis and inflammation.

#### Myriocin improves diet-induced atherosclerosis

**Fig 7:**
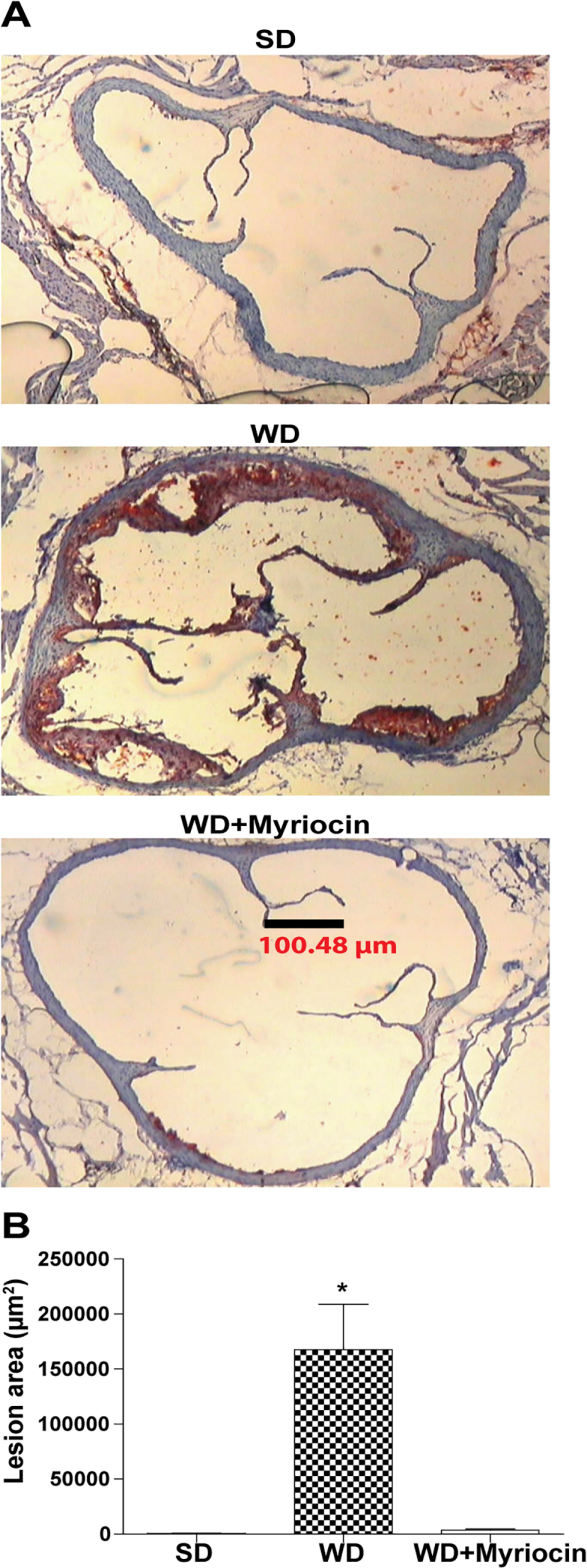
Myriocin reduces atherosclerosis in LDLR^-/-^ mice fed a WD (mean±SEM, n = 5/group). Representative formalin fixed serial sections of the aortic root prepared using cryostat, stained with Oil-Red-O **(A)**. Lesion areas were quantified and expressed as total plug area **(B)**. *P<0.001 compared to the SD and WD + Myriocin groups.

The WD, but not the SD resulted in substantial aortic root lesions as assessed by Oil Red-O staining. Myriocin supplementation significantly reduced aortic root atherosclerosis by ~50 fold, compared with a WD-fed mice (**Fig 7A and 7B**). Combined together with the liver histology data, these results indicate that pharmacological inhibition of ceramide production prevents diet-induced NAFLD and associated atherosclerosis.

#### Expression of genes involved in intestinal fat and cholesterol absorption

**Fig 8:**
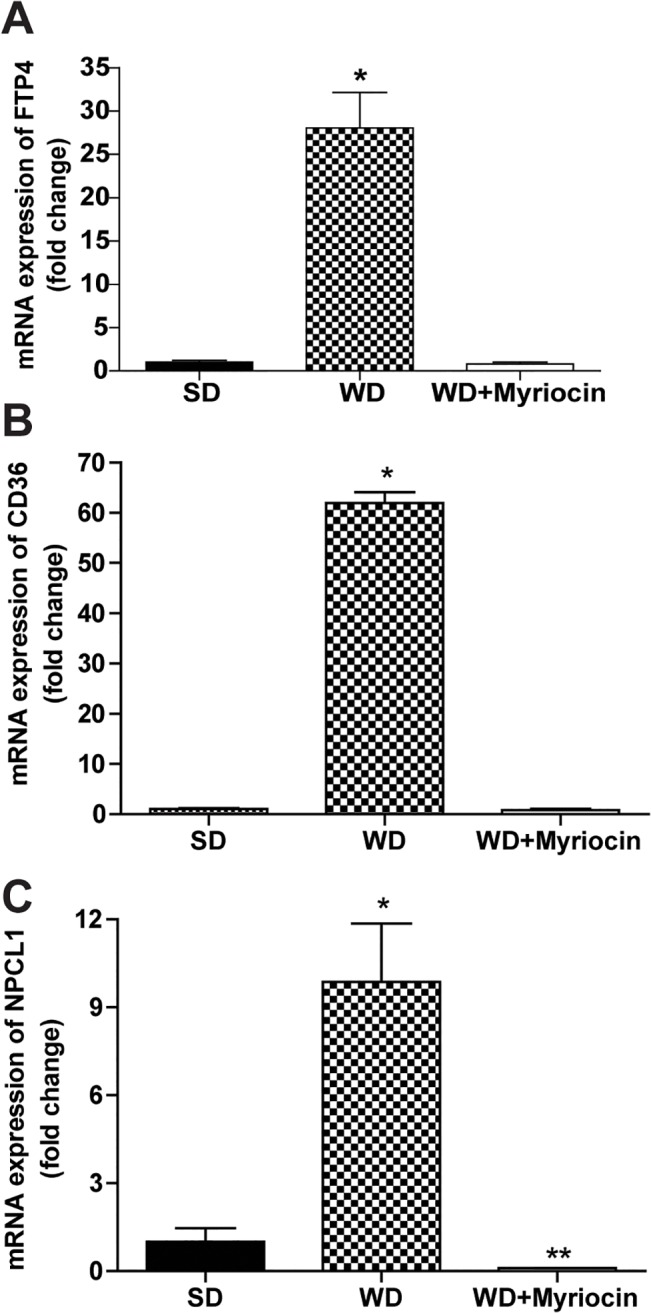
Effect of a WD and myriocin on the mRNA expression of intestinal genes (normalized relative to 18S) involved in cholesterol and fatty acid absorption. FATP4 **(A)**, CD36 (**B)** and NPCL1 **(C)**. *P<0.05 compared to the SD and WD+Myriocin groups. **P<0.05 significantly different from the SD group.

In order to understand the mechanisms of ceramide-induced NAFLD and atherosclerosis, we quantified the intestinal expression of genes involved in fat and cholesterol absorption. The WD caused more than a 30-fold increase in expression of mRNA fatty acid transfer protein 4 (FATP4) and CD36, proteins involved in intestinal fatty acid absorption (**Fig 8A and 8B**). Intestinal mRNA expression of Niemann-Pick C1-Like 1 (NPC1L1) (**Fig 8C**), a gene responsible for intestinal cholesterol uptake, was also significantly increased. Myriocin significantly suppressed the WD induced intestinal expression of these genes (**Fig 8A–8C**) suggesting that reduced intestinal cholesterol and fatty acid uptake may be an indirect result of systemic ceramide inhibition.

#### Effect of diet and inhibition of ceramide on hepatic lipid metabolism

**Hepatic fatty acid synthesis**: To determine whether in the presence of dietary oversupply of fatty acids *de novo* fatty acid synthesis contributes to hepatic steatosis, we also quantified hepatic lipogenesis using heavy water tracer. A WD increased both hepatic total palmitate content (14.5±0.9 vs. 10.7±0.8 mg/g liver, *P*<0.01) and *de novo* synthesized palmitate (92.7±1.9 vs. 58.7±1.3 mg /g liver/day, *P*<0.05) relative to the SD-group. Myriocin also reduced total palmitate content compared to both WD and SD groups, due to its dramatic reduction of hepatic palmitate synthesis (27.89±1.15 mg/g liver/day, *P*<0.0001 vs. both SD and WD). Thus, myriocin suppressed a WD-induced de novo lipogenesis (**S5 Fig**). This was consistent with the effect of myriocin on hepatic expression of genes involved in lipogenesis **(S6 Fig)**: SREBP1c, fatty acid synthase (FAS), stearoyl-CoA desaturase1 (SCD1), PPARγ, which were all upregulated by the WD and downregulated by myriocin.

Similar changes occurred in hepatic expression of genes involved in triglyceride metabolism. The WD increased the expression of DGAT1 and DGAT2 approximately 80% and hepatic lipase (HL) by 9-fold, but caused no change in lipoprotein lipase (LPL). Myriocin prevented WD-induced alterations in mRNA expression of genes involved in triglyceride metabolism (**S7 Fig**). These results indicate that despite dietary oversupply of fatty acids, *de novo* lipogenesis was increased in this LDLR^-/-^ mouse model of NAFLD and atherosclerosis.

**Hepatic cholesterol synthesis:** To assess the effect of insulin resistance on hepatic cholesterol metabolism in the presence of dietary cholesterol supply, we also quantified hepatic cholesterol flux. The WD diet caused a 4-fold increase in hepatic cholesterol content. Due to dietary oversupply of cholesterol in the WD, the hepatic PR of cholesterol was ~15-fold lower in the WD compared to the SD group. The kinetics data on hepatic cholesterol synthesis and content corresponded to the suppression of SREBP2 and HMG-CoA Reductase mRNA. Myriocin treatment improved WD-induced alterations in hepatic cholesterol metabolism (**S8 Fig**). Specifically, myriocin significantly reduced hepatic cholesterol accumulation, increased the cholesterol PR and partially upregulated the expression of SREBP2 and HMG-CoA Reductase compared to WD. This in part could be related to decreased absorption of dietary cholesterol as suggested by suppression of the genes involved in cholesterol absorption (**Fig 8**).

## Discussion

This study is the first to examine the role of sphingolipids in NAFLD and associated atherosclerosis. In the LDLR^-/-^ mouse model we have demonstrated that pharmacological inhibition of ceramide biosynthesis (i) reduces hepatic levels of toxic ceramide species, (ii) reduces plasma ceramides and SM involved in atherosclerosis, (iii) reduces plasma triglycerides, and total cholesterol, (iv) reduces ApoB but increases plasma ApoAI levels leading to a reduced ApoB/ApoAI ratio, (v) improves insulin sensitivity, (vi) reduces hepatic fat and cholesterol accumulation, inflammation and fibrosis and (vii) improves atherosclerosis. Our results show that these changes in part may be related to (i) reduced hepatic lipogenesis and ApoB production, (ii) increased HDL turnover and expression of genes involved in RCT (iii) and decreased absorption of dietary fatty acids and cholesterol. Overall, these results suggest that sphingolipids are key mediators of NAFLD and atherosclerosis.

Dyslipidemia and inflammation are common key factors in the pathogenesis of both NAFLD and atherosclerosis. Hepatic inflammation is also an independent predictor of endothelial dysfunction in patients with NASH [1,41]. The LDLR^-/-^ mouse model shares many phenotypic characteristics of NAFLD patients including diet-induced obesity, insulin resistance dyslipidemia and, increased CVD. The LDL receptor deficiency in these animals leads to hepatic inflammation, fibrosis [42] and accelerates atherosclerosis [43]. While hepatic steatosis can be induced in LDLR^-/-^ mice after 7 days on a WD [42], it takes 12 weeks to develop atherosclerosis in these animals, suggesting that in the setting of LDL deficiency NAFLD precedes atherosclerosis [44]. These studies suggest that hepatic steatosis and inflammation could enhance the risk for WD-induced atherosclerosis in NAFLD. However, the molecular mediators linking NAFLD and atherosclerosis are unknown.

Ceramide has been postulated as a primary lipid mediator of insulin resistance and inflammation in different organs [45,46]. Ceramide and ceramide-derived sphingolipids induce insulin resistance by altering multiple steps in the insulin signaling pathway, including membrane translocation of insulin receptor substrate 1 (IRS-1) and protein phosphatase 2A (PP2A) mediated dephosphorylation of Akt; a key mediator of insulin signaling [13]. In this study we used a mass spectrometry based targeted lipidomics approach to study the effect of WD and myriocin on hepatic ceramide metabolism, which enabled us to assess changes in different ceramide species and total ceramides. Distinct ceramide species are synthesized in mammals by a family of six ceramide synthases (CerS1-6) [47]. C16:0 and C24:0 ceramides are generated by CerS5/6 and CerS2, respectively. The acyl chain length defines different functions of distinct ceramide species. For example, overexpression of CerS5 promotes apoptosis while overexpression of CerS2 protects from apoptosis. In addition, CerS2 deficiency in mice results in severe hepatopathy with an increased rate of hepatic apoptosis from ~30 days of age and progressive hepatomegaly and hepatocellular carcinoma from ~10 months of age. These data suggest that C24:0 ceramide plays a critical role in liver homeostasis [48]. On the other hand, CerS6 deficiency-induced suppression of C16:0 ceramide in mice protects against insulin resistance [46]. In contrast, the increase of C16:0 ceramide by overexpression of CerS6 in isolated primary hepatocytes results in triglyceride accumulation, inhibition of insulin signaling and altered mitochondrial function; all characteristics observed in NAFLD [49]. Finally, it has been demonstrated that liver-specific ablation of CerS2 in mice causes hepatic insulin resistance due to reduced phosphorylation of the insulin receptor and its inability to translocate into detergent-resistant membranes [50]. This was associated with altered plasma membrane composition due to reduced C24:0 ceramide levels. Collectively, these findings highlight causal roles for both C16:0 and C24:0 ceramides in insulin resistance and hepatic homeostasis.

Importantly, we found that a WD leads to increased levels of C16:0 but reduced levels of C24:0 in the liver. It is expected that the high palmitate content of a WD and insulin resistance-induced lipogenesis lead to increased production of C16:0 ceramide. However, it is unclear why C24:0 ceramide was reduced on a WD. One possible reason for these bidirectional changes in hepatic C16:0 and C24:0 content may be related to sequestration of a sphingoid precursor with excessive palmitoyl-CoA that results in limited precursor availability for C24:0 ceramide synthesis. Published data suggest that the levels of long-chain C16:0 and very-long-chain ceramide C24:0 are also reciprocally regulated. In particular, it has been demonstrated that mice fed a high-fat diet display increased C16:0 but decreased C24:0 ceramide in white adipose and liver tissues [46] that were associated with decreased CerS2, but increased CerS6 activities. In addition, CerS2 haploinsufficiency in mice leads to compensatory increase in long-chain C16:0 ceramide that confers susceptibility to diet-induced insulin resistance and dysregulated lipid metabolism [49]. Consistent with these data, we found that the impact of a WD on ceramide metabolism is associated with altered hepatic phosphorylation of Akt (S473). Notably, myriocin-induced sensitization of the liver to insulin, coincided with increased Akt phosphorylation and normalized levels of ceramide species (reduced C16:0 ceramide and increased C24:0 ceramide). Because of the critical roles for CerS6 and CerS2 in liver homeostasis and insulin sensitivity [48], and cell membrane integrity [50], changes in C16:0 and C24:0 ceramide observed in our study may be involved in insulin resistance and development of NAFLD in LDLR^-/-^ mice.

The current animal model also shares histologic abnormalities in the liver found in NAFLD patients including steatosis, lobular inflammation, centrilobular sinusoidal fibrosis and apoptosis; all of which can be explained in part by ceramide induced injury. Recently it has been demonstrated that ceramides play a key role in hepatic triglyceride accumulation and inflammation [49,51]. Our study expands on these findings and suggests that ceramides are also involved in hepatic oxidative stress, fibrosis and apoptosis. Our flux studies suggest that the role of ceramides in hepatic steatosis is related to increased hepatic lipogenesis as discussed above. Ceramides and other sphingolipids are also known to be pro-inflammatory, to induce apoptosis via caspase dependent and independent pathways, and to be fibrogenic [52].

Increased hepatic and plasma triglycerides and cholesterol resulting from the WD were reversed by myriocin **(Table 1).** This was not related to increased fatty acid oxidation (data not presented), but rather to reduced lipogenesis by myriocin, which was associated with the decrease in both ApoB production and triglyceride secretion, indicating reduced VLDL secretion. A similar inhibitory effect of myriocin on hepatic triglyceride production via suppression of hepatic SREBP-1c was recently reported in a diet-induced hamster model of insulin resistance [53].

We also examined the role of ceramide in the pathogenesis of atherosclerosis in NAFLD. Significant increases in hepatic expression of HL and LPL genes with a WD suggest that an increase in VLDL conversion to LDL production in LDLR^-/-^ mice contribute to the development of atherosclerosis. The increased plasma ApoB levels with a WD and their reduction with myriocin confirm that change in ApoB production was mainly driven by hepatic triglyceride synthesis. Myriocin treatment increased HDL flux, increased hepatic expression of genes involved in RCT and almost completely abolished atherosclerosis induced by a WD. These data suggest that myriocin affects both HDL biogenesis and functionality, including RCT. These results are also in agreement with the recent finding that depletion of the ceramide metabolite SM in cell membrane promotes cholesterol efflux [54], the key step in RCT. In the current study, inhibition of ceramide and SM biosynthesis by myriocin also was associated with improved plasma lipids (i.e. reduced ceramide, SM, total cholesterol, TG and increased HDLc) and lipoproteins (i.e. increased ApoAI, reduced both ApoB and ApoB/ApoAI ratio) and significantly protected against atherosclerosis. Based on these results we postulate that regulation of lipid and lipoprotein metabolism by ceramides could be one of the possible mechanisms that link diet-induced NAFLD and atherosclerosis.

In addition to its role in hepatic lipid and lipoprotein metabolism, circulating ceramide may also promote atherosclerosis through direct effects on endothelial function [55]. It was recently shown that liver-derived ceramides along with triglycerides and cholesterol are packaged with VLDL and released into the circulation [45]. Lipidomics analysis of different lipoprotein fractions demonstrated that indeed, plasma ceramides are concentrated in VLDL and LDL particles [56]. Ceramide-loaded LDL has been shown to stimulate TNF-α and IL-6 production in cultured macrophages through NF-κB activation [45] suggesting that ceramide-induced cytokine production in the circulation can target endothelial cells, which would exacerbate atherosclerosis through inflammatory mechanisms. In addition, increased ceramide and SM content in oxidized remnant lipoproteins may enhance susceptibility to aggregation and retention in arterial walls, as atherosclerotic lesions in humans have a 10–50 fold increase in ceramide content compared to plasma LDLs [57,58]. Finally, ceramides in circulation can cause endothelial dysfunction through reduced NO bioavailability. Consistent with this hypothesis, it was recently shown that pharmacological inhibition of ceramide biosynthesis with myriocin, and heterozygous deletion of dihydroceramide desaturase improves vascular reactivity and hypertension in diet-induced obese mice *in vivo*, suggesting that ceramides are involved in endothelial dysfunction [55]. The mechanistic *in vitro* experiments with palmitate incubation of endothelial cells revealed that this was related to ceramide-induced activation of PP2A, which in turn reduces endothelial nitric oxide synthase (eNOS) phosphorylation, the key step in eNOS activation required for NO synthesis. Thus, independent of effects on NAFLD, liver-derived ceramides may also directly contribute to atherosclerosis. While our results demonstrate the importance of hepatic production of ceramide in the pathogenesis of both NAFLD and atherosclerosis, future studies are warranted to investigate the mechanistic role of ceramides in temporal relationship between NAFLD onset and development of atherosclerosis.

Furthermore, our study suggest that the harmful effect of ceramide on NAFLD and atherosclerosis could be also in part relate to its role in the uptake of dietary cholesterol and fatty acids. It has been demonstrated that pharmacological inhibition or genetic deficiency of SPT, the rate limiting enzyme in sphingolipid biosynthesis, reduces cholesterol absorption [59]. Consistent with these results, we found that myriocin treatment reduced the expression of intestinal cholesterol transporter NPC1L1. These results are similar to that reported for ezetimibe, a well-known cholesterol reducing drug that inhibits intestinal cholesterol absorption through inhibition of NPC1L1 [60]. However, in contrast to ezetimibe, our data indicate that the effect of myrocin on intestinal uptake is not specific to cholesterol. Overexpression of intestinal CD36 and FATP4 fatty acid transporters by a WD and their suppression with myriocin suggest that sphingolipids are also involved in intestinal uptake of fatty acids. Mechanistically, CD36 and FATP4 mediate fatty acid dissociation from albumin and the accumulation of fatty acids at the outer leaflet of the cell membrane, followed by flip-flop across the phospholipid bilayer to the cytosolic site [61]. Previously it has been shown that SPT deficiency in mice results in reduced SM levels on the apical membranes of enterocytes [59] and depleting membrane SM with myriocin or sphingomyelinase reduces fatty acid uptake [62]. Thus, myriocin induced improvement in dyslipidemia and hepatic steatosis in our study may also be related to changes in NPC1L1, CD36 and FATP4 expression and alterations in lipid raft composition that reduce intestinal fatty acid and cholesterol uptake. Future studies of lipid metabolism with simultaneous quantification of both fatty acid/cholesterol absorption and lipogenesis/lipoprotein turnover in the same experiment would improve understanding of the effect of ceramides on NAFLD and atherosclerosis.

## Conclusion

We conclude that inhibition of ceramide biosynthesis protects against diet-induced NAFLD and atherosclerosis in LDLR^-/-^ mice by reducing insulin resistance, suppressing de novo lipogenesis, and enhancing HDL biogenesis and turnover. These findings emphasize the importance of ceramide in the pathogenesis of both NALFD and atherosclerosis.

## Supporting Information

S1 Fig Myriocin reverses a Western diet (WD) induced triglyceride secretion (mean ± SEM, n = 5/group). Overnight fasted mice were injected with tyloxapol, and triglyceride measured before and after tyloxapol injection (from 0 to 120 min) (**A**). Hepatic triglyceride secretion was determined as mg per deciliter per min (**B**). *P<0.05 compared to the WD group.
(DOC)

S2 Fig Time course ^2^H-labeling of total body water in LDLR^-/-^ mice. (DOC)

S3 Fig Myriocin reduces a WD induced hepatic triglyceride accumulation (mean ± SEM, n = 6). *P<0.05 compared to the SD and WD+Myriocin groups.
(DOC)

S4 Fig Myriocin does not attenuate a WD-induced hepatic oxidative stress in LDLR^-/-^ mice (mean ± SEM, n = 6). Glutathione redox ratio was calculated from the hepatic content of reduced GSH and oxidized GSSG. **P<0.05 significantly different from the WD and WD+Myriocin groups.
(DOC)

S5 Fig Myriocin suppresses a WD-induced *de novo* lipogenesis (mean ± SEM, n = 6). Hepatic palmitate content. (**A**) Hepatic palmitate production rate (**B**). *P<0.05 significantly different from the SD and WD+Myriocin groups. **P<0.05 significantly different from the SD group.
(DOC)

S6 Fig Effect of myriocin on hepatic mRNA expression of genes involved in lipogenesis measured by RT-PCR (mean ± SEM, n = 6). Expression of SREBP1c (**A**), FAS (**B**), SCD1 (**C**) and PPARγ (**D**) relative to 18S. *P<0.05 compared to the SD and WD+Myriocin groups. **P<0.05 significantly different from the SD group.
(DOC)

S7 Fig Myriocin prevents WD-induced alterations in mRNA expression of genes involved in triglyceride metabolism. Expression of DGAT1 (**A**), DGAT2 (**B**), HL (**C**), and LPL (**D**) relative to 18S (mean ± SEM, n = 6). *P<0.05 significantly different from the SD and WD + Myriocin groups. **P<0.05 significantly different from the WD group.
(DOC)

S8 Fig Myriocin treatment improves WD-induced alterations in hepatic cholesterol metabolism (mean ± SEM, n = 6). Hepatic cholesterol content (**A**), Hepatic cholesterol PR (**B**), mRNA expression of SREBP2 (**C**) and HMG-CoA Reductase (**D**). *P<0.05 significantly different from two other groups. **P<0.05 significantly different from the SD and WD groups.
(DOC)

S1 Table Composition of high fat diet containing cholesterol. (DOCX)

S2 Table List of primers used for qRT-PCR. (DOCX)

## References

[1] Alkhouri N, Tamimi TA, Yerian L, Lopez R, Zein NN, Feldstein AE. (2010) The inflamed liver and atherosclerosis: a link between histologic severity of nonalcoholic fatty liver disease and increased cardiovascular risk. Dig Dis Sci 55: 2644–2650. 10.1007/s10620-009-1075-y 19960252

[2] Targher G, Bertolini L, Padovani R, Zoppini G, Zenari L, Falezza G. (2005) Associations between liver histology and carotid intima-media thickness in patients with nonalcoholic fatty liver disease. Arterioscler Thromb Vasc Biol 25: 2687–2688. 16306438 10.1161/01.ATV.0000189299.61568.79

[3] Parekh S, Anania FA (2007) Abnormal lipid and glucose metabolism in obesity: implications for nonalcoholic fatty liver disease. Gastroenterology 132: 2191–2207. 17498512 10.1053/j.gastro.2007.03.055

[4] Brown MS, Goldstein JL (1986) A receptor-mediated pathway for cholesterol homeostasis. Science 232: 34–47. 3513311 10.1126/science.3513311

[5] Rye KA, Bursill CA, Lambert G, Tabet F, Barter PJ (2009) The metabolism and anti-atherogenic properties of HDL. J Lipid Res 50 Suppl: S195–200. 10.1194/jlr.R800034-JLR200 19033213 PMC2674714

[6] Walldius G, Jungner I, Aastveit AH, Holme I, Furberg CD, Sniderman AD (2004) The apoB/apoA-I ratio is better than the cholesterol ratios to estimate the balance between plasma proatherogenic and antiatherogenic lipoproteins and to predict coronary risk. Clin Chem Lab Med 42: 1355–1363. 15576296 10.1515/CCLM.2004.254

[7] Chavez JA, Siddique MM, Wang ST, Ching J, Shayman JA, Summers SA (2014) Ceramides and Glucosylceramides are independent antagonists of insulin signaling. J Biol Chem 289: 723–34. 10.1074/jbc.M113.522847 24214972 PMC3887200

[8] Bikman BT (2012) A role for sphingolipids in the pathophysiology of obesity-induced inflammation. Cell Mol Life Sci 69: 2135–2146. 10.1007/s00018-012-0917-5 22294100 PMC11114706

[9] Maceyka M, Spiegel S (2014) Sphingolipid metabolites in inflammatory disease. Nature 510: 58–67. 10.1038/nature13475 24899305 PMC4320971

[10] Pagadala M, Kasumov T, McCullough AJ, Zein NN, Kirwan JP (2012) Role of ceramides in nonalcoholic fatty liver disease. Trends Endocrinol Metab 23: 365–371. 10.1016/j.tem.2012.04.005 22609053 PMC3408814

[11] Haus JM, Kashyap SR, Kasumov T, Zhang R, Kelly KR, Defronzo RA, et al (2009) Plasma ceramides are elevated in obese subjects with type 2 diabetes and correlate with the severity of insulin resistance. Diabetes 58: 337–343. 10.2337/db08-1228 19008343 PMC2628606

[12] Huang H, Kasumov T, Gatmaitan P, Heneghan HM, Kashyap SR, Schauer PR, et al (2011) Gastric bypass surgery reduces plasma ceramide subspecies and improves insulin sensitivity in severely obese patients. Obesity (Silver Spring) 19: 2235–2240. 10.1038/oby.2011.107 21546935 PMC3809956

[13] Summers SA (2010) Sphingolipids and insulin resistance: the five Ws. Curr Opin Lipidol 21: 128–135. 10.1097/MOL.0b013e3283373b66 20216312

[14] Jiang XC, Paultre F, Pearson TA, Reed RG, Francis CK, Lin M, et al (2000) Plasma sphingomyelin level as a risk factor for coronary artery disease. Arterioscler Thromb Vasc Biol 20: 2614–2618. 11116061 10.1161/01.atv.20.12.2614

[15] Fujita T, Inoue K, Yamamoto S, Ikumoto T, Sasaki S, Toyama R, et al (1994) Fungal metabolites. Part 11. A potent immunosuppressive activity found in Isaria sinclairii metabolite. J Antibiot (Tokyo) 47: 208–215. 8150717 10.7164/antibiotics.47.208

[16] Park TS, Panek RL, Mueller SB, Hanselman JC, Rosebury WS, Robertson AW, et al (2004) Inhibition of sphingomyelin synthesis reduces atherogenesis in apolipoprotein E-knockout mice. Circulation 110: 3465–3471. 15545514 10.1161/01.CIR.0000148370.60535.22

[17] Hojjati MR, Li Z, Zhou H, Tang S, Huan C, Ooi E, et al (2005) Effect of myriocin on plasma sphingolipid metabolism and atherosclerosis in apoE-deficient mice. J Biol Chem 280: 10284–10289. 15590644 10.1074/jbc.M412348200

[18] Park TS, Panek RL, Rekhter MD, Mueller SB, Rosebury WS, Robertson A, et al (2006) Modulation of lipoprotein metabolism by inhibition of sphingomyelin synthesis in ApoE knockout mice. Atherosclerosis 189: 264–272. 16458317 10.1016/j.atherosclerosis.2005.12.029

[19] Kasumov T, Willard B, Li L, Li M, Conger H, Buffa JA, et al (2013) 2H2O-based high-density lipoprotein turnover method for the assessment of dynamic high-density lipoprotein function in mice. Arterioscler Thromb Vasc Biol 33: 1994–2003. 10.1161/ATVBAHA.113.301700 23766259 PMC3958961

[20] Heneghan HM, Huang H, Kashyap SR, Gornik HL, McCullough AJ, Schauer PR, et al (2013) Reduced cardiovascular risk after bariatric surgery is linked to plasma ceramides, apolipoprotein-B100, and ApoB100/A1 ratio. Surg Obes Relat Dis 9: 100–107. 10.1016/j.soard.2011.11.018 22264909 PMC3337956

[21] Bieghs V, Van Gorp PJ, Wouters K, Hendrikx T, Gijbels MJ, Lutjohann D, et al (2012) LDL receptor knock-out mice are a physiological model particularly vulnerable to study the onset of inflammation in non-alcoholic fatty liver disease. PLoS One 7: e30668 10.1371/journal.pone.0030668 22295101 PMC3266276

[22] Wu L, Vikramadithyan R, Yu S, Pau C, Hu Y, Goldberg IJ, et al (2006) Addition of dietary fat to cholesterol in the diets of LDL receptor knockout mice: effects on plasma insulin, lipoproteins, and atherosclerosis. J Lipid Res 47: 2215–2222. 16840797 10.1194/jlr.M600146-JLR200

[23] Subramanian S, Goodspeed L, Wang S, Kim J, Zeng L, Ioannou GN, et al (2011) Dietary cholesterol exacerbates hepatic steatosis and inflammation in obese LDL receptor-deficient mice. J Lipid Res 52: 1626–1635. 10.1194/jlr.M016246 21690266 PMC3151683

[24] Gupte AA, Liu JZ, Ren Y, Minze LJ, Wiles JR, Collins AR, et al (2010) Rosiglitazone attenuates age- and diet-associated nonalcoholic steatohepatitis in male low-density lipoprotein receptor knockout mice. Hepatology (Baltimore, Md) 52: 2001–2011. 10.1002/hep.23941 20938947 PMC2991614

[25] Joven J, Rull A, Ferrâe N, Escoláa-Gil JC, Marsillach J, Coll B, et al (2007) The results in rodent models of atherosclerosis are not interchangeable: the influence of diet and strain. Atherosclerosis 195: e85–92. 17651742 10.1016/j.atherosclerosis.2007.06.012

[26] Deevska GM, Rozenova KA, Giltiay NV, Chambers MA, White J, Boyanovsky BB, et al (2009) Acid Sphingomyelinase Deficiency Prevents Diet-induced Hepatic Triacylglycerol Accumulation and Hyperglycemia in Mice. The Journal of biological chemistry 284: 8359–8368. 10.1074/jbc.M807800200 19074137 PMC2659194

[27] Teupser D, Persky AD, Breslow JL (2003) Induction of atherosclerosis by low-fat, semisynthetic diets in LDL receptor-deficient C57BL/6J and FVB/NJ mice: comparison of lesions of the aortic root, brachiocephalic artery, and whole aorta (en face measurement). Arteriosclerosis, thrombosis, and vascular biology 23: 1907–1913. 12907460 10.1161/01.ATV.0000090126.34881.B1

[28] Goldberg IJ, Hu Y, Noh HL, Wei J, Huggins LA, Rackmill MG, et al (2008) Decreased lipoprotein clearance is responsible for increased cholesterol in LDL receptor knockout mice with streptozotocin-induced diabetes. Diabetes 57: 1674–1682. 10.2337/db08-0083 18346984

[29] Brunengraber DZ, McCabe BJ, Kasumov T, Alexander JC, Chandramouli V, Previs SF (2003) Influence of diet on the modeling of adipose tissue triglycerides during growth. Am J Physiol Endocrinol Metab 285: E917–925. 12799315 10.1152/ajpendo.00128.2003

[30] Li L, Willard B, Rachdaoui N, Kirwan JP, Sadygov RG, Stanley WC, et al (2012) Plasma proteome dynamics: analysis of lipoproteins and acute phase response proteins with 2H2O metabolic labeling. Mol Cell Proteomics 11: M111 014209. 10.1074/mcp.M111.014209 PMC3394944 22393261

[31] Siri P, Candela N, Zhang YL, Ko C, Eusufzai S, Ginsberg HN, et al (2001) Post-transcriptional stimulation of the assembly and secretion of triglyceride-rich apolipoprotein B lipoproteins in a mouse with selective deficiency of brown adipose tissue, obesity, and insulin resistance. J Biol Chem 276: 46064–46072. 11598138 10.1074/jbc.M108909200

[32] Kasumov T, Huang H, Chung YM, Zhang R, McCullough AJ, Kirwan JP (2010) Quantification of ceramide species in biological samples by liquid chromatography electrospray ionization tandem mass spectrometry. Anal Biochem 401: 154–161. 10.1016/j.ab.2010.02.023 20178771 PMC2872137

[33] Kasumov T, Sharma N, Huang H, Kombu RS, Cendrowski A, Stanley WC, et al (2009) Dipropionylcysteine ethyl ester compensates for loss of citric acid cycle intermediates during post ischemia reperfusion in the pig heart. Cardiovasc Drugs Ther 23: 459–469. 10.1007/s10557-009-6208-1 19967553 PMC2873150

[34] Shah V, Herath K, Previs SF, Hubbard BK, Roddy TP (2010) Headspace analyses of acetone: a rapid method for measuring the 2H-labeling of water. Anal Biochem 404: 235–237. 10.1016/j.ab.2010.05.010 20488158

[35] Bligh EG, Dyer WJ (1959) A rapid method of total lipid extraction and purification. Can J Biochem Physiol 37: 911–917. 13671378 10.1139/o59-099

[36] Previs SF, Mahsut A, Kulick A, Dunn K, Andrews-Kelly G, Johnson C, et al (2011) Quantifying cholesterol synthesis in vivo using (2)H(2)O: enabling back-to-back studies in the same subject. J Lipid Res 52: 1420–1428. 10.1194/jlr.D014993 21498887 PMC3122914

[37] Baglione J, Smith JD (2006) Quantitative assay for mouse atherosclerosis in the aortic root. Methods Mol Med 129: 83–95. 17085806 10.1385/1-59745-213-0:83

[38] Xue B, Kim YB, Lee A, Toschi E, Bonner-Weir S, Kahn CR, et al (2007) Protein-tyrosine phosphatase 1B deficiency reduces insulin resistance and the diabetic phenotype in mice with polygenic insulin resistance. J Biol Chem 282: 23829–23840. 17545163 10.1074/jbc.M609680200

[39] Deevska GM, Sunkara M, Morris AJ, Nikolova-Karakashian MN (2012) Characterization of secretory sphingomyelinase activity, lipoprotein sphingolipid content and LDL aggregation in ldlr-/- mice fed on a high-fat diet. Biosci Rep 32: 479–490. 10.1042/BSR20120036 22712892 PMC3475451

[40] Zhou H, Li W, Wang SP, Mendoza V, Rosa R, Hubert J, et al (2012) Quantifying apoprotein synthesis in rodents: coupling LC-MS/MS analyses with the administration of labeled water. J Lipid Res 53: 1223–1231. 10.1194/jlr.D021295 22389331 PMC3351829

[41] Targher G, Bertolini L, Padovani R, Zenari L, Zoppini G, Falezza G (2004) Relation of nonalcoholic hepatic steatosis to early carotid atherosclerosis in healthy men: role of visceral fat accumulation. Diabetes Care 27: 2498–2500. 15451925 10.2337/diacare.27.10.2498

[42] Wouters K, van Gorp PJ, Bieghs V, Gijbels MJ, Duimel H, Lütjohann D, et al (2008) Dietary cholesterol, rather than liver steatosis, leads to hepatic inflammation in hyperlipidemic mouse models of nonalcoholic steatohepatitis. Hepatology 48: 474–486. 10.1002/hep.22363 18666236

[43] Collins AR, Lyon CJ, Xia X, Liu JZ, Tangirala RK, Yin F, et al (2009) Age-accelerated atherosclerosis correlates with failure to upregulate antioxidant genes. Circ Res 104: e42–54. 10.1161/CIRCRESAHA.108.188771 19265038

[44] Teupser D, Persky AD, Breslow JL (2003) Induction of atherosclerosis by low-fat, semisynthetic diets in LDL receptor-deficient C57BL/6J and FVB/NJ mice: comparison of lesions of the aortic root, brachiocephalic artery, and whole aorta (en face measurement). Arterioscler Thromb Vasc Biol 23: 1907–1913. 12907460 10.1161/01.ATV.0000090126.34881.B1

[45] Boon J, Hoy AJ, Stark R, Brown RD, Meex RC, Henstridge DC, et al (2013) Ceramides contained in LDL are elevated in type 2 diabetes and promote inflammation and skeletal muscle insulin resistance. Diabetes 62: 401–410. 10.2337/db12-0686 23139352 PMC3554351

[46] Turpin SM, Nicholls HT, Willmes DM, Mourier A, Brodesser S, Wunderlich CM, et al (2014) Obesity-induced CerS6-dependent C16:0 ceramide production promotes weight gain and glucose intolerance. Cell Metab 20: 678–686. 10.1016/j.cmet.2014.08.002 25295788

[47] Levy M, Futerman AH (2010) Mammalian ceramide synthases. IUBMB Life 62: 347–356. 10.1002/iub.319 20222015 PMC2858252

[48] Pewzner-Jung Y, Brenner O, Braun S, Laviad EL, Ben-Dor S, Feldmesser E, et al (2010) A critical role for ceramide synthase 2 in liver homeostasis: II. insights into molecular changes leading to hepatopathy. J Biol Chem 285: 10911–10923. 10.1074/jbc.M109.077610 20110366 PMC2856297

[49] Raichur S, Wang ST, Chan PW, Li Y, Ching J, Chaurasia B, et al (2014) CerS2 haploinsufficiency inhibits beta-oxidation and confers susceptibility to diet-induced steatohepatitis and insulin resistance. Cell Metab 20: 687–695. 10.1016/j.cmet.2014.09.015 25295789

[50] Park JW, Park WJ, Kuperman Y, Boura-Halfon S, Pewzner-Jung Y, Futerman AH. (2013) Ablation of very long acyl chain sphingolipids causes hepatic insulin resistance in mice due to altered detergent-resistant membranes. Hepatology 57: 525–532. 10.1002/hep.26015 22911490

[51] Jiang C, Xie C, Li F, Zhang L, Nichols RG, Krausz KW, et al (2015) Intestinal farnesoid X receptor signaling promotes nonalcoholic fatty liver disease. J Clin Invest 125: 386–402. 10.1172/JCI76738 25500885 PMC4382255

[52] Scarpa MC, Baraldo S, Marian E, Turato G, Calabrese F, Saetta M, et al (2013) Ceramide expression and cell homeostasis in chronic obstructive pulmonary disease. Respiration 85: 342–349. 10.1159/000341185 23018286

[53] Dekker MJ, Baker C, Naples M, Samsoondar J, Zhang R, Qiu W, et al (2013) Inhibition of sphingolipid synthesis improves dyslipidemia in the diet-induced hamster model of insulin resistance: evidence for the role of sphingosine and sphinganine in hepatic VLDL-apoB100 overproduction. Atherosclerosis 228: 98–109. 10.1016/j.atherosclerosis.2013.01.041 23466071

[54] Gulshan K, Brubaker G, Wang S, Hazen SL, Smith JD (2013) Sphingomyelin depletion impairs anionic phospholipid inward translocation and induces cholesterol efflux. J Biol Chem 288: 37166–37179. 10.1074/jbc.M113.512244 24220029 PMC3873571

[55] Zhang QJ, Holland WL, Wilson L, Tanner JM, Kearns D, Cahoon JM, et al (2012) Ceramide mediates vascular dysfunction in diet-induced obesity by PP2A-mediated dephosphorylation of the eNOS-Akt complex. Diabetes 61: 1848–1859. 10.2337/db11-1399 22586587 PMC3379648

[56] Wiesner P, Leidl K, Boettcher A, Schmitz G, Liebisch G (2009) Lipid profiling of FPLC-separated lipoprotein fractions by electrospray ionization tandem mass spectrometry. J Lipid Res 50: 574–585. 10.1194/jlr.D800028-JLR200 18832345

[57] Rapp JH, Lespine A, Hamilton RL, Colyvas N, Chaumeton AH, Tweedie-Hardman J, et al (1994) Triglyceride-rich lipoproteins isolated by selected-affinity anti-apolipoprotein B immunosorption from human atherosclerotic plaque. Arteriosclerosis and thrombosis: a journal of vascular biology / American Heart Association 14: 1767–1774. 10.1161/01.atv.14.11.1767 7947602

[58] Hoff HF, O'Neil J, Pepin JM, Cole TB (1990) Macrophage uptake of cholesterol-containing particles derived from LDL and isolated from atherosclerotic lesions. European heart journal 11: 105–115. 2226518 10.1093/eurheartj/11.suppl_e.105

[59] Li Z, Park TS, Li Y, Pan X, Iqbal J, Lu D, et al (2009) Serine palmitoyltransferase (SPT) deficient mice absorb less cholesterol. Biochim Biophys Acta 1791: 297–306. 10.1016/j.bbalip.2009.01.010 19416652 PMC4371774

[60] Garcia-Calvo M, Lisnock J, Bull HG, Hawes BE, Burnett DA, Braun MP, et al (2005) The target of ezetimibe is Niemann-Pick C1-Like 1 (NPC1L1). Proc Natl Acad Sci U S A 102: 8132–8137. 15928087 10.1073/pnas.0500269102 PMC1149415

[61] Abumurad NA, Sfeir Z (2000) Lipid transporters: membrane transport systems for cholesterol and fatty acids. Curr Opin Clin Nutr Metab Care 3: 255–262. 10929670 10.1097/00075197-200007000-00003

[62] Ehehalt R, Sparla R, Kulaksiz H, Herrmann T, Fullekrug J, Stremmel W (2008) Uptake of long chain fatty acids is regulated by dynamic interaction of FAT/CD36 with cholesterol/sphingolipid enriched microdomains (lipid rafts). BMC Cell Biol 9: 45 10.1186/1471-2121-9-45 18700980 PMC2533316

