# Supplementary figures and images for: Ceramide as a Mediator of Non-Alcoholic Fatty Liver Disease and Associated Atherosclerosis
Source: PLoS One. 2015 May 20;10(5):e0126910. doi: 10.1371/journal.pone.0126910 (PMC4439060; doi:10.1371/journal.pone.0126910)

**S1 Fig**.


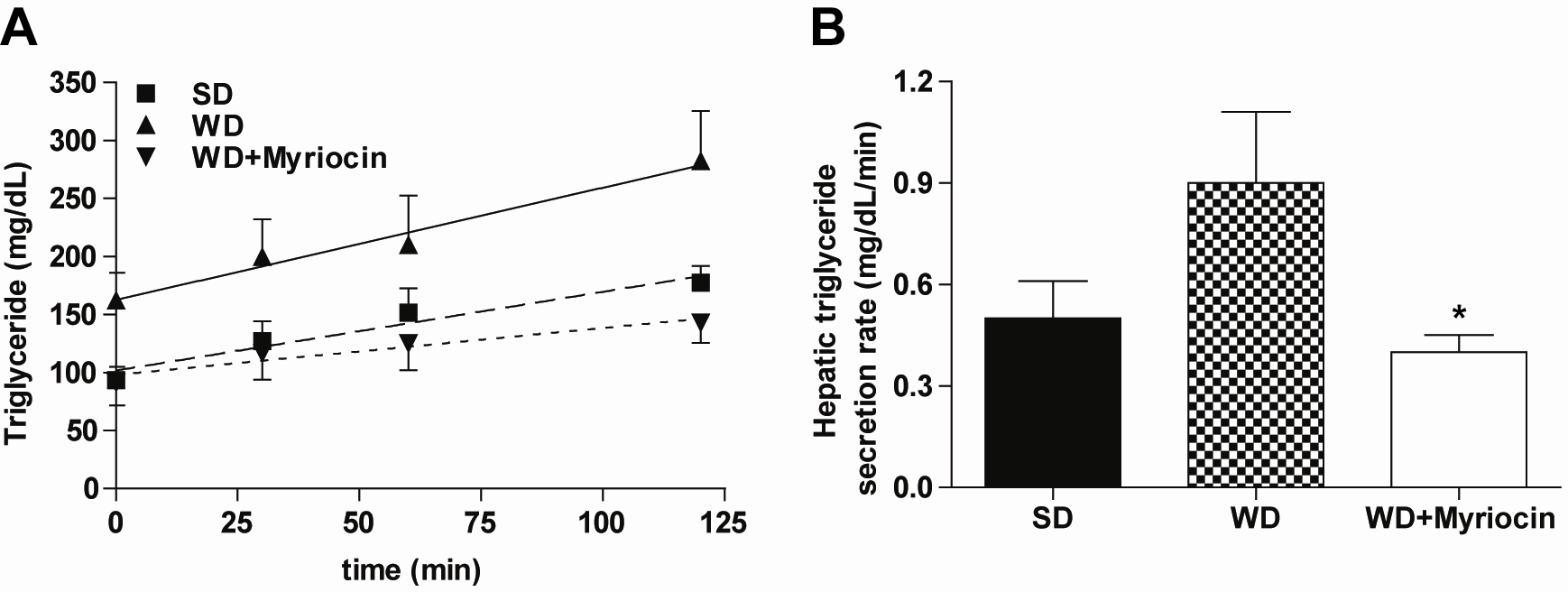

Supplement: S1 Fig — Overnight fasted mice were injected with tyloxapol, and triglyceride measured before and after tyloxapol injection (from 0 to 120 min) (A). Hepatic triglyceride secretion was determined as mg per deciliter per min (B). *P<0.05 compared to the WD group. (DOC) [file pone.0126910.s001.doc]

**S2 Fig**.


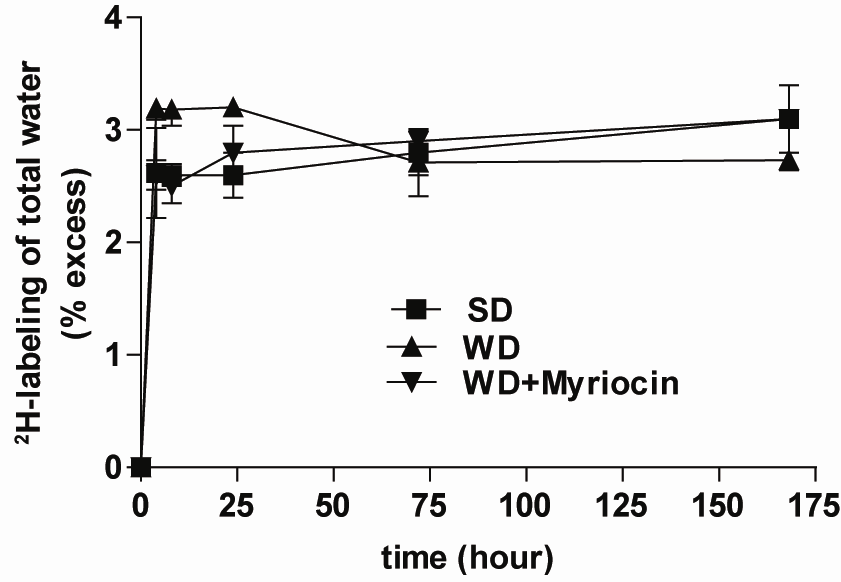

Supplement: S2 Fig — (DOC) [file pone.0126910.s002.doc]

**S3 Fig.**


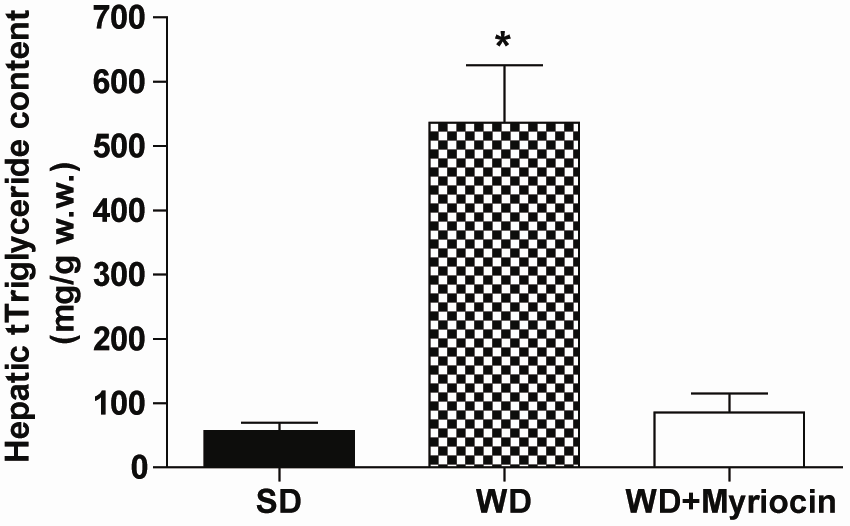

Supplement: S3 Fig — *P<0.05 compared to the SD and WD+Myriocin groups. (DOC) [file pone.0126910.s003.doc]

**S4 Fig**.


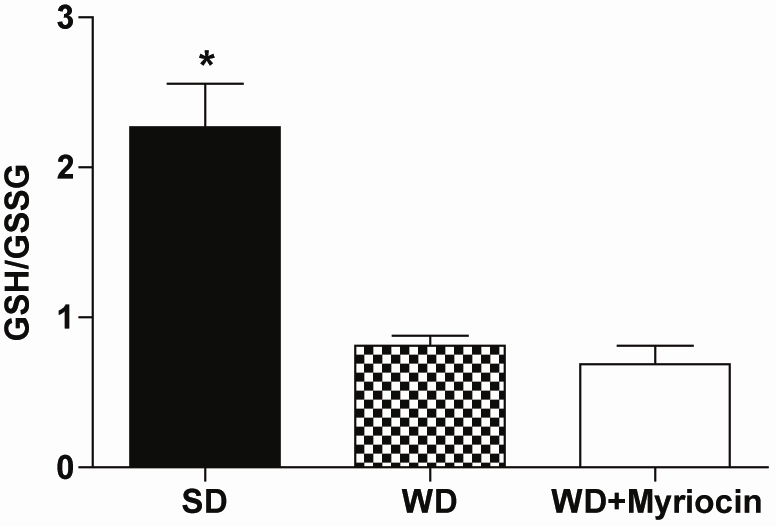

Supplement: S4 Fig — Glutathione redox ratio was calculated from the hepatic content of reduced GSH and oxidized GSSG. **P<0.05 significantly different from the WD and WD+Myriocin groups. (DOC) [file pone.0126910.s004.doc]

**S5 Fig.**


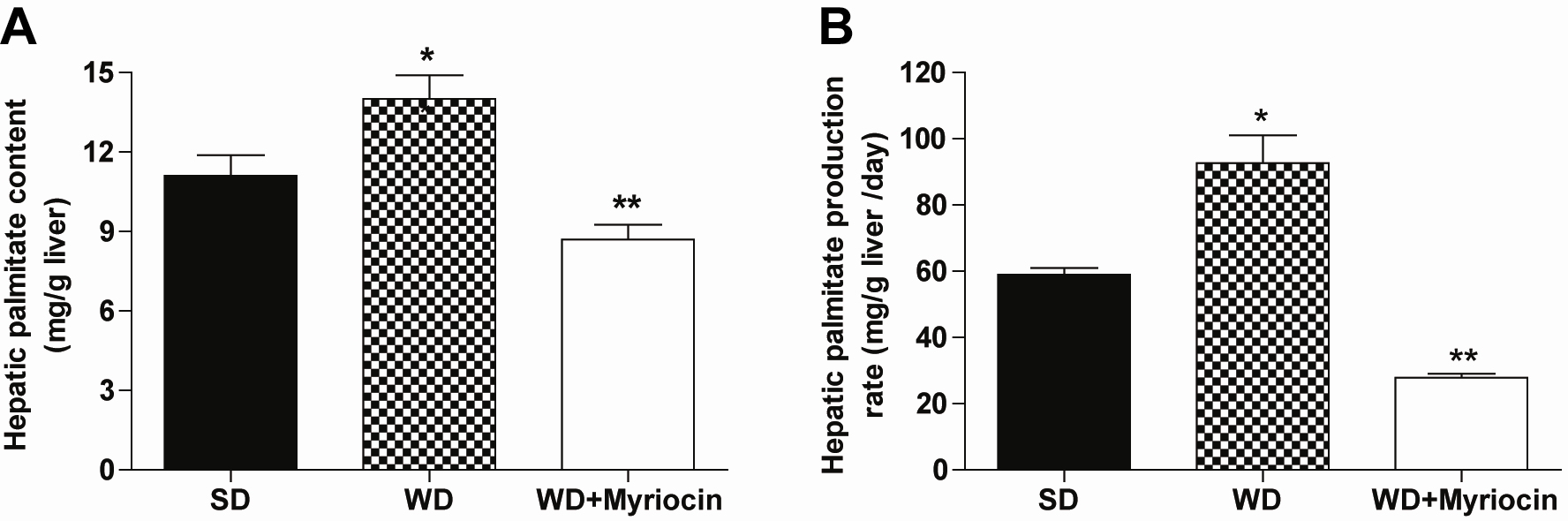

Supplement: S5 Fig — (A) Hepatic palmitate production rate (B). *P<0.05 significantly different from the SD and WD+Myriocin groups. **P<0.05 significantly different from the SD group. (DOC) [file pone.0126910.s005.doc]

**S6 Fig**.


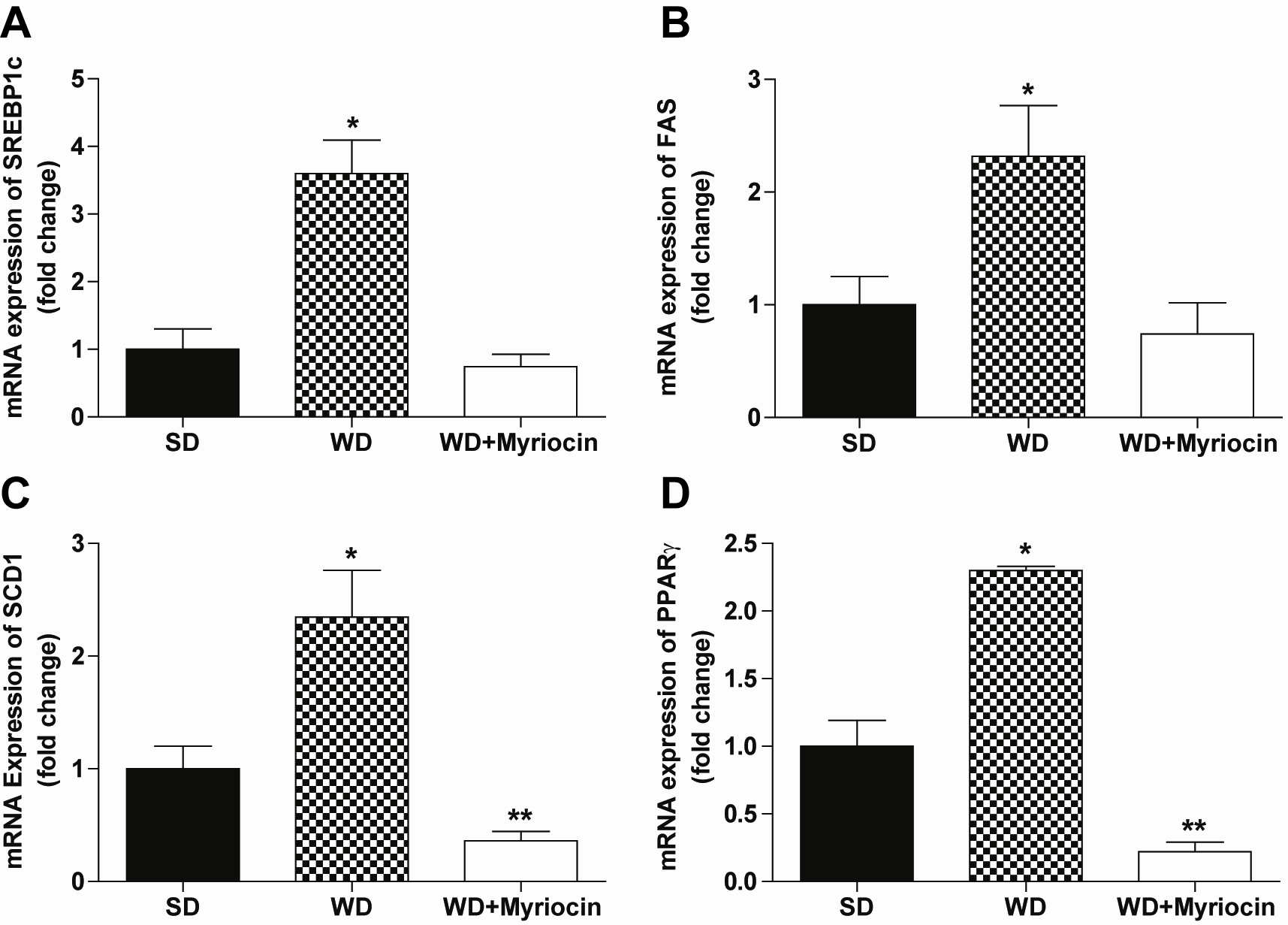

Supplement: S6 Fig — Expression of SREBP1c (A), FAS (B), SCD1 (C) and PPARγ (D) relative to 18S. *P<0.05 compared to the SD and WD+Myriocin groups. **P<0.05 significantly different from the SD group. (DOC) [file pone.0126910.s006.doc]

**S7 Fig**.


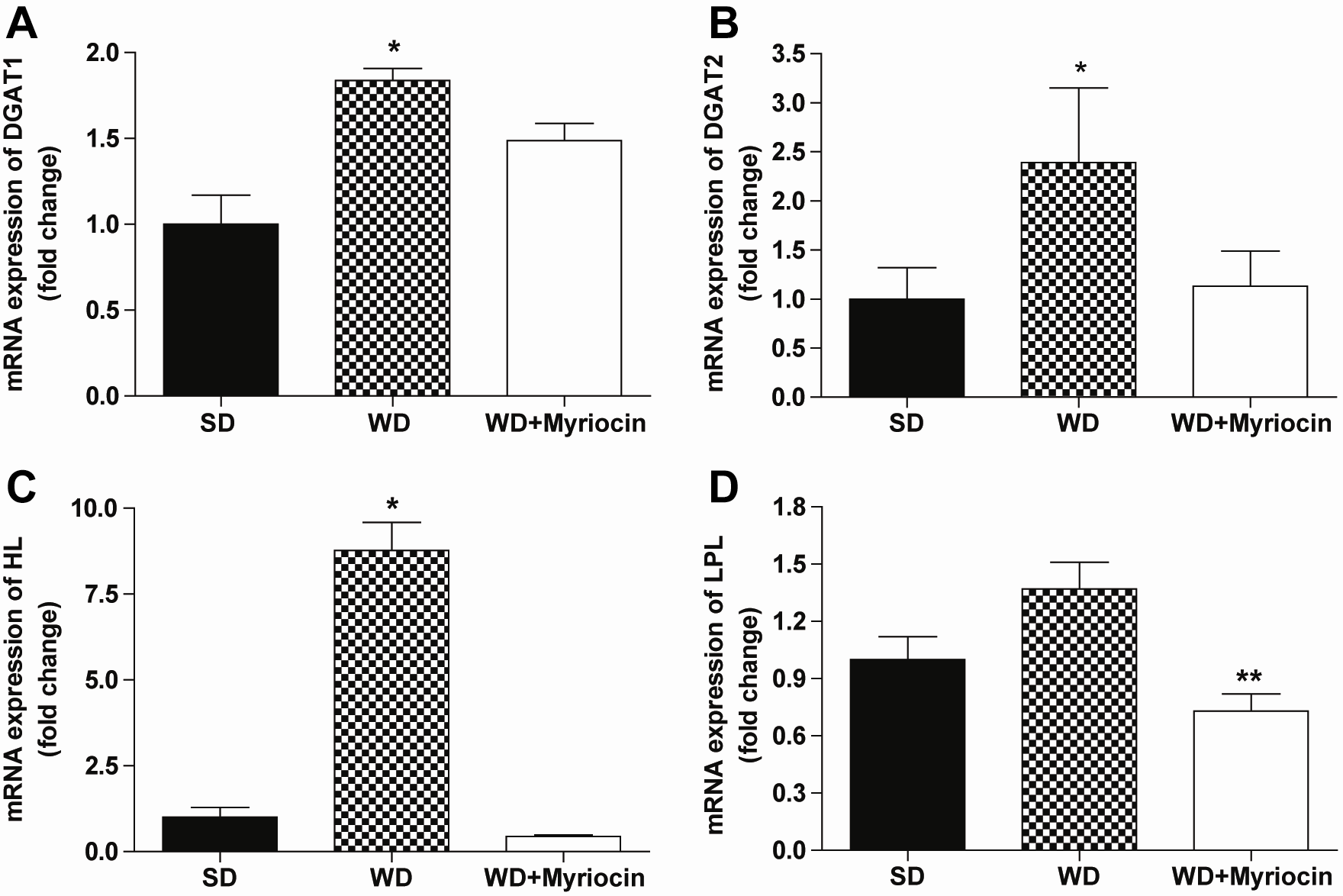

Supplement: S7 Fig — Expression of DGAT1 (A), DGAT2 (B), HL (C), and LPL (D) relative to 18S (mean ± SEM, n = 6). *P<0.05 significantly different from the SD and WD + Myriocin groups. **P<0.05 significantly different from the WD group. (DOC) [file pone.0126910.s007.doc]

**S8 Fig.**


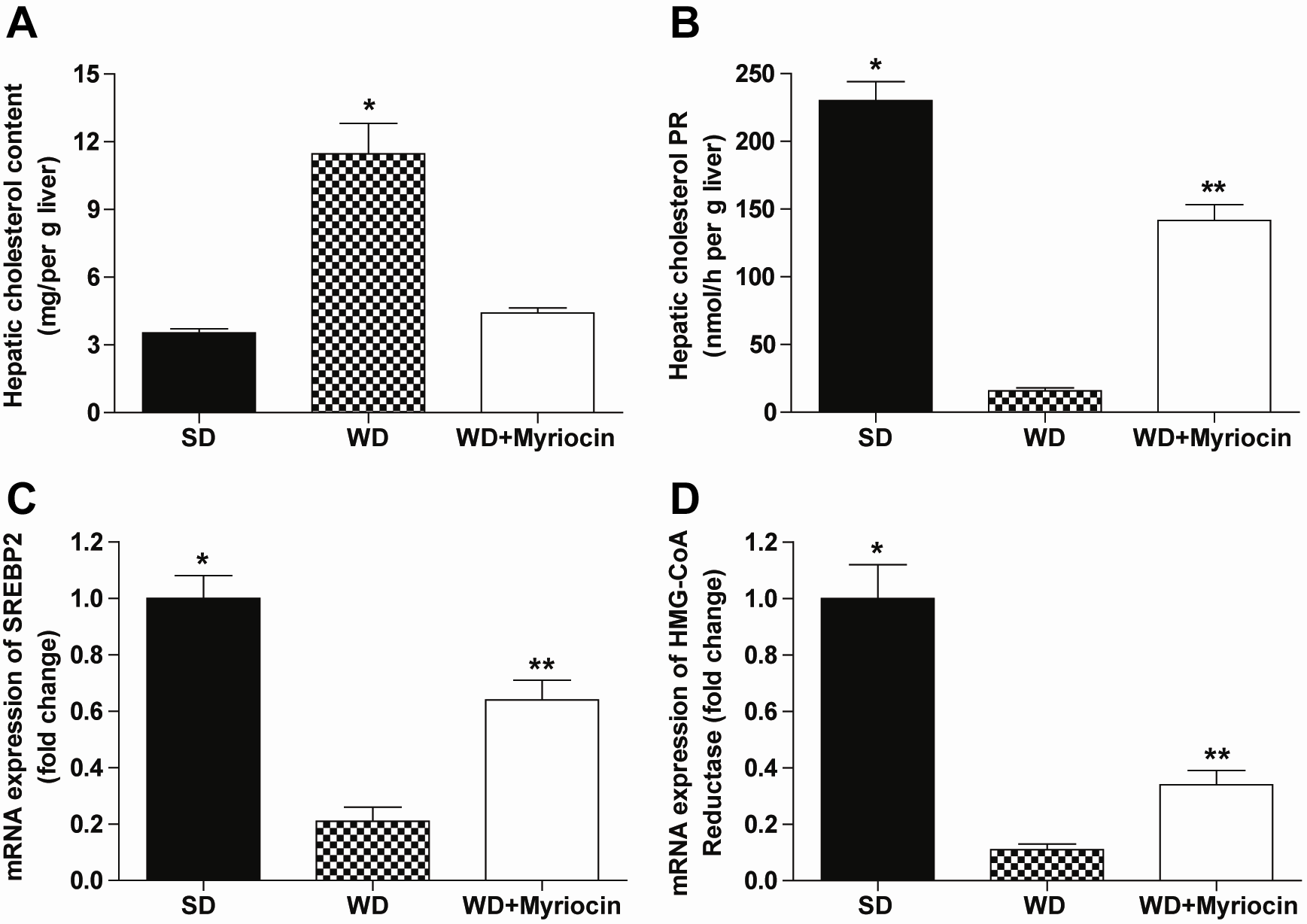

Supplement: S8 Fig — Hepatic cholesterol content (A), Hepatic cholesterol PR (B), mRNA expression of SREBP2 (C) and HMG-CoA Reductase (D). *P<0.05 significantly different from two other groups. **P<0.05 significantly different from the SD and WD groups. (DOC) [file pone.0126910.s008.doc]
