# Supplementary material for: Ceramide as a Mediator of Non-Alcoholic Fatty Liver Disease and Associated Atherosclerosis
Source: PLoS One. 2015 May 20;10(5):e0126910. doi: 10.1371/journal.pone.0126910 (PMC4439060; doi:10.1371/journal.pone.0126910)
Supplement: S1 Table — (DOCX) [file pone.0126910.s009.docx]

**S1Table. Composition of high fat diet containing cholesterol.**

|  | **gram cal%** | **Kcal%** |
| --- | --- | --- |
| **Fat** | 23.5 | 45 |
| **Carbohydrate** | 41.3 | 35 |
| **Protein** | 23.6 | 20 |
| **Ingredients** | **gm** | **kcal** |
| **Lard** | 177.5 | 1597.5 |
| **Soybean Oil** | 25 | 225 |
| **Corn starch** | 72.8 | 291.2 |
| **Maltodextrin 10** | 100 | 400 |
| **Sucrose** | 172.8 | 691.2 |
| **Casein, 80 Mesh** | 200 | 800 |
| **Added Cholesterol (gm)** | 2.1 |  |
| **Added Cholesterol (%)** | 0.24 |  |
