# Supplementary material for: Ceramide as a Mediator of Non-Alcoholic Fatty Liver Disease and Associated Atherosclerosis
Source: PLoS One. 2015 May 20;10(5):e0126910. doi: 10.1371/journal.pone.0126910 (PMC4439060; doi:10.1371/journal.pone.0126910)
Supplement: S2 Table — (DOCX) [file pone.0126910.s010.docx]

**S2 Table. List of primers used for qRT-PCR.**

| **Genes** | **Mouse Primers** |
| --- | --- |
| mSrb1 Forward | ATG GGC CAG CGT GCT TTT ATG AAC |
| mSrb1 Reverse | ACG CCC GTG AAG ACA GTG AAG ACC |
| HMGCoA Reductase Forward | CCT GGG CCC CAC ATT CA |
| HMGCoA Reductase Reverse | GAC ATG GTG CCA ACT CCA ATC |
| SREBP1a/Forward | GGC CGA GAT GTG CGA ACT |
| SREBP1a/Reverse | TTG TTG ATG AGC TGG AGC ATG T |
| SREBP2/Forward | GCG TTC TGG AGA CCA TGG |
| SREBP2/Reverse | ACA AAG TTG CTC TGA AAA CAA ATC A |
| ApoAI forward | ACG TAT GGC AGC AAG ATG AAC |
| ApoAI Reverse | AGA GCT CCA CAT CCT CTT TCC |
| PPAR/Gamma-Forward | ATA AAG CAT CAG GCT TCC ACT |
| PPAR/Gamma-Reverse | GCA CTT CTG AAA CCG ACA GTA |
| ABCA1/forward | GGT TTG GAG ATG GTT ATA CAA TAG TTG T |
| ABCA1/Reverse | TTC CCG GAA ACG CAA GTC |
| ABCG5/Forward | TGG CCC TGC TCA GCA TCT |
| ABCG5/Reverse | ATT TTT AAA GGA ATG GGC ATC TCT T |
| ABCG8/Forward | CCG TCG TCA GAT TTC CAA TGA |
| ABCG8/Reverse | GGC TTC CGA CCC ATG AAT G |
| NPC1L1/Forward | GAG AGC CAA AGA TGC TAC TAT CTT CA |
| NPC1L1/Reverse | CCC GGG AAG TTG GTC ATG |
| SCD-1/forward | TAC TAC AAG CCC GGC CTC C |
| SCD-1/Reverse | CAG CAG TAC CAG GGC ACC A |
| IL-6/Forward | AGTTGCCTTCTTGGGACTGA |
| IL-6/Reverse | CAGAATTGCCATTGCACAAC |
| Col1a1/Forward | GAGCGGAGAGTACTGGATCG |
| Col1a1/Reverse | GCTTCTTTTCCTTGGGGTTC |
| TNF-alpha/Forward | TATGGCTCAGGGTCCAACTC |
| TNF-alpha/Reverse | CTCCCTTTGCAGAACTCAGG |
| FAS/forward | TTGCTGGCACTACAGAATGC |
| FAS/reverse | AACAGCCTCAGAGCGACAAT |
|  |  |
